# Lateralized costs of divided attention to faces

**DOI:** 10.3758/s13414-025-03189-1

**Published:** 2025-12-28

**Authors:** Samantha C. Lee, Lars Strother

**Affiliations:** Department of Psychology at the University of Nevada, Reno, 1664 N. Virginia St, Reno, NV 89557 USA

**Keywords:** Face perception, Face sex, Dual-task, Divided attention, Laterality, LVF advantage

## Abstract

We used a dual-task paradigm to study the relationship between lateralized face perception and attention by measuring the costs of dividing attention between faces viewed in opposite visual fields. Observers performed judgments of either the sex, orientation, or color of one (single-task, cued) or both (dual-task, uncued) faces in a tachistoscopically viewed pair. We observed dual-task costs (i.e., decreased accuracy for dual- relative to single-task performance) for categorical judgments of face sex (female/male) and orientation (upright/inverted), both of which necessitated visual processing of face-specific information. We did not observe costs for judgments of face color (red-tinted/greyscale), which could be performed without processing face-specific visual information per se. We also observed an unexpected “feature contrast” effect of category-incongruency for judgments of face sex, such that observers showed no dual-task cost when faces in a pair belonged to opposite categories (e.g., female/male) as compared with the same category (e.g., female/female). Finally, dual-task costs for judgments of face sex and orientation (but not color) showed a left visual field (LVF) advantage: dual-task costs were greater in the right visual field (RVF) than in the LVF. We interpret this LVF cost advantage for judgments of face sex and orientation as indicative of the type of visual processing needed to perform face-based judgments. Our results show, for the first time, that the LVF advantage in face perception is directly related to capacity limits induced by divided attention to faces.

**Supplementary Information:**

The online version contains supplementary material available at 10.3758/s13414-025-03189-1.

Face perception and attention are among the most widely studied topics in psychology and cognitive neuroscience. A considerable amount of research on face perception concerns the potentially “special” (Farah et al., 1998) status of faces as visual stimuli, but relatively little research has focused on whether face perception shows visual processing capacity privileges or not. Certain kinds of face perception have been shown to exhibit limits in the number of faces that can be processed in parallel, but there is not yet consensus concerning the stage of face processing at which such limits occur (e.g., Fitousi, 2021; Qarooni et al., 2022). In this study, we investigated face processing capacity limits in the context of *divided attention* (Broadbent, 1958)—the ability to allocate visual processing resources simultaneously to multiple spatial locations, stimulus features, or objects. Despite decades of research on face perception and attention studied separately, our scientific understanding of how different kinds of face perception interact with attention remains incomplete (but see review by Palermo & Rhodes, 2007). Our understanding of the relationship between lateralized face processing and the potential costs of dividing attention between faces is even more limited, and the main focus of the current study. Cerebral laterality is directly related to the allocation of visual attention (Heilman & Abell, 1980; Kinsbourne, 1970; Reuter-Lorenz et al., 1990), and is therefore essential to a complete understanding of the relationship between face perception and divided attention.

One starting point from which to summarize what is known about the role of attention in face perception is to consider whether attention is necessary for face perception or not. Studies of individual face perception that did not require observers to divide attention between multiple faces have yielded mixed results. There is some evidence that certain kinds of face processing (e.g., “holistic”) require attention (e.g., Crist, 2008; Reinitz et al., 1994), but other findings suggest otherwise (e.g., Boutet et al., 2002; Lavie et al., 2003; Norman & Tokarev, 2014). Additional studies by Reddy et al. (2004, 2006) found that observers can perceive the sex and identity of a face without any cost of performing a concurrent attention-demanding visual task on a non-face stimulus. In contrast to the mixed results concerning the necessity of attention for single-face perception, the perception of multiple faces appears to involve more consistent evidence for the necessity of attention, and consequent limits with respect to the number of faces that can be processed in parallel, even for basic perceptual judgments of faces.

Palermo and Rhodes (2002) used a *dual-task* divided attention paradigm to show capacity limits for the holistic processing of face identity (Pashler, 1994). In their study, observers were presented with target and flanker faces and performed different tasks on each. They found that when each of the two kinds of tasks involved holistic processing, processing of the target face was disrupted, and thus concluded that the number of faces that can be processed holistically at a time is limited. To our knowledge, the study by Palermo and Rhodes is unique in its application of a dual-task divided attention paradigm to study prospective capacity limits in the perception of multiple faces. Indeed, there have been surprisingly few studies of multiface processing capacity limits in general (see brief overviews in Bindemann et al., 2005; Qarooni et al., 2022; also see Haberman & Whitney, 2007, 2009, for overview of perceiving summary statistics from multiface “ensemble” stimuli)—and few if any attempts to relate face processing capacity to lateralized face perception. Findings from selective attention studies that measured target–distractor interference (i.e., the extent to which task-irrelevant stimuli are processed obligatorily; Young et al., 1986) suggest capacity limits at different stages of face processing. Consistent with the findings of Palermo and Rhodes, studies by Bindemann and colleagues have shown capacity limitations that limit the perception of face identity (Bindemann et al., 2005, 2007), which they characterize as reliant on “cognitively deep,” relatively late stages of face processing (Qarooni et al., 2022). Intriguingly, Bindemann and colleagues have also reported findings consistent with a processing capacity bottleneck at early, “cognitively shallow” stages of face processing, including for the perception of face sex (Bindemann et al., 2005), but after visual processing underlying face detection (Qarooni et al., 2022; but see Fitousi, 2021)—which is fast and efficient, and entails configural processing (Maurer et al., 2002; Palermo & Rhodes, 2007; Piepers & Robbins, 2012).

Despite evidence that capacity limits can occur during the early stages of face processing, the possibility remains that such limits would not generalize to a paradigm in which observers performed the same task on more than one face. The capacity limits reported by Palermo and Rhodes (2002) could have at least partially reflected the performance of different judgments on target versus flanker faces, and capacity limits observed in target-distractor interference paradigms are not necessarily generalizable to dual-task divided attention paradigms. Therefore, of central interest in our study was whether dual-task costs necessarily occur when the same judgment is performed on two faces (dual-task) as compared with one (single-task). Several recent divided attention studies have used this approach to study capacity limits in the perception of non-face objects (Campbell et al., 2024; Popovkina et al., 2021, 2023; White et al., 2018, 2020), and to show that multi-item capacity limits are absent or negligible when visual processing of basic feature information (e.g., color) is sufficient to perform a given task (Harrison et al., 2022; White et al., 2018, 2020). This approach has not yet, however, been used to study dual-task costs for face perception.

We tested for evidence of dual-task costs on the performance of two *face-based* judgments—binary categorical judgments of face sex^1^ (female/male) and orientation (upright/inverted), both of which could be performed with unfamiliar faces—and on judgments of a basic visual feature (color) that could be performed without face processing per se. Our interest in capacity limits for judgments of face sex stems from the finding of Bindemann et al. (2005), mentioned earlier, that perception of face sex is limited to one face at a time (i.e., all-or-none serial) even though face sex information is processed relatively early. From a very young age (Quinn et al., 2019), human observers spontaneously and accurately perceive face sex categorically, even in the absence of perceptually salient nameable sex-specific cues (Bruce et al., 1993; Gandolfo & Downing, 2023; Hügelschäfer et al., 2016; Rakić et al., 2018; Rekow et al., 2020; Tomelleri & Castelli, 2012). Given ongoing disagreement concerning the degree to which the perception of face sex relies on holistic versus feature-based processing (Baudouin & Humphreys, 2006; Brown & Perrett, 1993; Bruce et al., 1993; Schyns et al., 2002; Yamaguchi et al., 2013; Yokoyama et al., 2014; Zhao & Hayward, 2010), and concerning divided attention costs for holistic face perception but not necessarily for face features (Palermo & Rhodes, 2002), we tested for evidence of dual-task costs consistent with all-or-none serial processing of face sex and the possibility that face feature information might preclude such extreme capacity limits. Face orientation judgments are also face-based but might be less likely than face sex judgments to show dual-task costs because orientation judgments do not require processing of identity-related or social trait information and can be made solely on the basis of first-order configural information (Maurer et al., 2002; Piepers & Robbins, 2012). Importantly, our main goal was to test for a potential relationship between lateralized face perception and divided attention, regardless of whether face-based tasks show cost-related differences or not.

None of the studies we described earlier that investigated capacity limits in multiface perception considered the potential relationship between capacity limits and lateralized face processing. Face perception is associated with right hemisphere superiority and a corresponding left visual field (LVF) advantage (Harrison & Strother, 2020, 2021; Yovel et al., 2003). We therefore leveraged the logic of the *divided field paradigm* (Bourne, 2006)—which hinges on a contralateral hemifield-hemisphere correspondence—in combination with our dual-task paradigm in order to test for hemifield asymmetries indicative of the recruitment of right-lateralized face processing resources. Attention is typically neglected (or at least not commonly manipulated) in divided field studies of lateralized face perception, which means the results of our study will have implications for the relationship, if any, between divided attention and the LVF advantage in face perception. Right hemisphere superiority and the LVF advantage in face perception are associated with visual processing of global (e.g., holistic or configural) face information versus spatially local stimulus features (Cattaneo et al., 2014; Hillger & Koenig, 1991; Parkin & Williamson, 1987; Rhodes, 1985; Ross & Turkewitz, 1981; Rossion et al., 2000; Scott & Nelson, 2006; Sergent, 1982, 1985; Sergent & Bindra, 1981). Therefore, of primary interest in the current study was whether prospective costs of dividing attention show an LVF advantage depending on the type of visual processing involved in face-based versus feature-based perceptual judgments. To test this possibility, we combined the general method of White et al. (2018, 2020) and Popovkina et al. (2021) with the logic of the divided field paradigm to:

1. Characterize dual-task costs relative to quantitative models of processing capacity; and
2. Test for LVF–RVF differences in dual-task cost and a prospective LVF cost advantage.

## Method

### Participants

Power analyses reported in previous studies that used a similar method (White et al., 2018, 2020; Popovkina et al., 2021) found that eight participants were sufficient to distinguish accuracy differences related to dual-task costs. Since we collected fewer trials per observer, we conducted a power analysis (assuming α = 0.05 and β = 0.05, for power of 95%) using G*Power (Version 3.1; Faul et al., 2009) on preliminary results obtained in our lab. Results of this power analysis showed that we would need eight participants to detect dual-task costs and distinguish between the all-or-none serial and the fixed-capacity parallel models, and ≥40 participants to detect dual-task hemifield asymmetries between the left visual field (LVF) and right visual field (RVF). We collected more in anticipation of excluding participants for eye movements or performance at ceiling or chance in the cued, single-task condition. The main reason we used this approach was because our participants received university course credit using a system that does not guarantee participants returning for more than one session.

Seventy-six volunteers participated in the main experiment (62 women and 14 men; mean age = 19.1 years); data from 47 of these volunteers were included in our primary analyses (see Results for details concerning the implementation of exclusion criteria). Sixty-nine additional volunteers (41 women, 25 men, and three nonbinary; mean age = 20.2 years) were recruited for a set of control analyses; data from 24 of these volunteers were included in these analyses (see Supplemental Materials, S.9., for further details). Each participant had normal or corrected-to-normal vision and was right-handed according to the Edinburgh Handedness Inventory (Oldfield, 1971). Volunteers were undergraduate students at the University of Nevada, Reno, and were granted course credit for participation. The experimental protocol adhered to the Declaration of Helsinki, and prior to participating, each observer provided informed consent according to the guidelines of the Institutional Review Board of the University of Nevada, Reno.

### Experimental design

Using a within-subjects design, we manipulated two independent variables (*cue* and *judgment* type) and collected accuracy data and confidence ratings as our dependent variables (later combined into a bias-free measure; see Results). As in several recent studies that used a dual-task paradigm to study divided attention to non-face objects (Campbell et al., 2024; Popovkina et al., 2021, 2023; White et al., 2018, 2020), we employed two *cue* conditions: a single-task condition in which observers judged only one of two simultaneously viewed faces (based on an exogenous visual cue to one face *location*, either LVF or RVF) and a dual-task condition in which observers performed judgments on both faces sequentially. Observers performed three different types of *judgment*, each with two target categories: judgments of face sex (female/male), orientation (upright/inverted), and color (red-tinted/greyscale).

### Stimuli and apparatus

Stimuli were selected from the Psychological Image Collection at Stirling database (PICS; pics.stir.ac.uk). We used 40 face stimuli (20 female; see Fig. S1) that were chosen to be the least ambiguous in terms of sex based on ratings from six independent observers who categorized each face as either female or male. The final set of faces was chosen based on interobserver agreement (see Supplemental Materials, S.1., for details). Faces were forward-facing with neutral expressions, and cropped using an oval aperture to remove external facial features (hair, ears, face shape, etc.) beyond the forehead, chin, and cheeks. Face stimuli were greyscale and equated for mean luminance using the SHINE toolbox in MATLAB (Willenbockel et al., 2010; The MathWorks Inc., Natick, MA). Stimuli were presented using the Psychophysics Toolbox (Brainard, 1997) for MATLAB on an Acer XFA240 monitor with 1,920 × 1,080-pixel resolution and a 120-Hz refresh rate. The face stimuli were resized to 120 × 130 pixels and subtended 3.5° × 3.7° visual angle (participants were seated approximately 56 cm from the monitor with a chin rest to maintain viewing distance throughout the experiment). Pairs of faces were displayed simultaneously, one in the LVF and one in the RVF, and were centered 3.2° from fixation on either side (precise location was held constant for faces in each hemifield). Faces in a pair always had different identities. The dynamic pre- and postmasks were each composed of three rapidly presented (22 ms each) checkerboard-scrambled faces of the same size as the face stimuli and presented in the same precise locations. Stimuli were presented on a white background and were centered around a grey fixation cross that was flanked by grey or cyan cue bars.

**Fig. 1:**
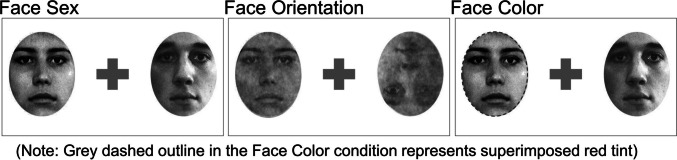
Judgment-specific stimulus manipulations. Physical properties of the face stimuli differed for each *judgment* type. For sex judgments, face pairs were greyscale, luminance-matched upright faces (full set shown in Fig. S1). For orientation judgments, the same face stimuli were masked to reduce visibility and were shown either upright or inverted (see text for details). For the color judgment, the same face stimuli were upright and were shown either red-tinted or greyscale (as in White et al., 2018). Note that the grey dashed outline in the Face Color panel represents superimposed red tint (see Fig. S1 for an example of superimposed color)

As shown in Fig. 1, physical properties of the face stimuli were different for each type of *judgment*. In the sex judgment trials, faces were presented as upright, greyscale images (as shown in Fig. S1). In the orientation judgment trials, the same faces were superimposed with a Gaussian noise mask to obscure visibility, and the face in each visual field had an independent 50% chance to be rotated 180 degrees and presented upside-down. For the color judgment trials, the same faces as those shown in Figure S1 were presented upright and each had a 50% chance of being tinted red (i.e., superimposed with a semi-transparent red mask). The opacity of the Gaussian noise masks (in the orientation judgment condition) and of the superimposed red masks (in the color judgment condition) was independently adjusted for each participant prior to the main experiment (see Supplemental Materials, S.4., for details). The purpose of the masks used for the orientation and color judgment face stimuli was similar but not identical. For the orientation judgment face stimuli, masks were used to decrease face visibility and thus avoid a ceiling effect in accuracy (as observed in preliminary pilot data). For the color judgment face stimuli, we used the red-tinted masks for the same purpose as White et al. (2018).

### Procedure

**Fig. 2:**
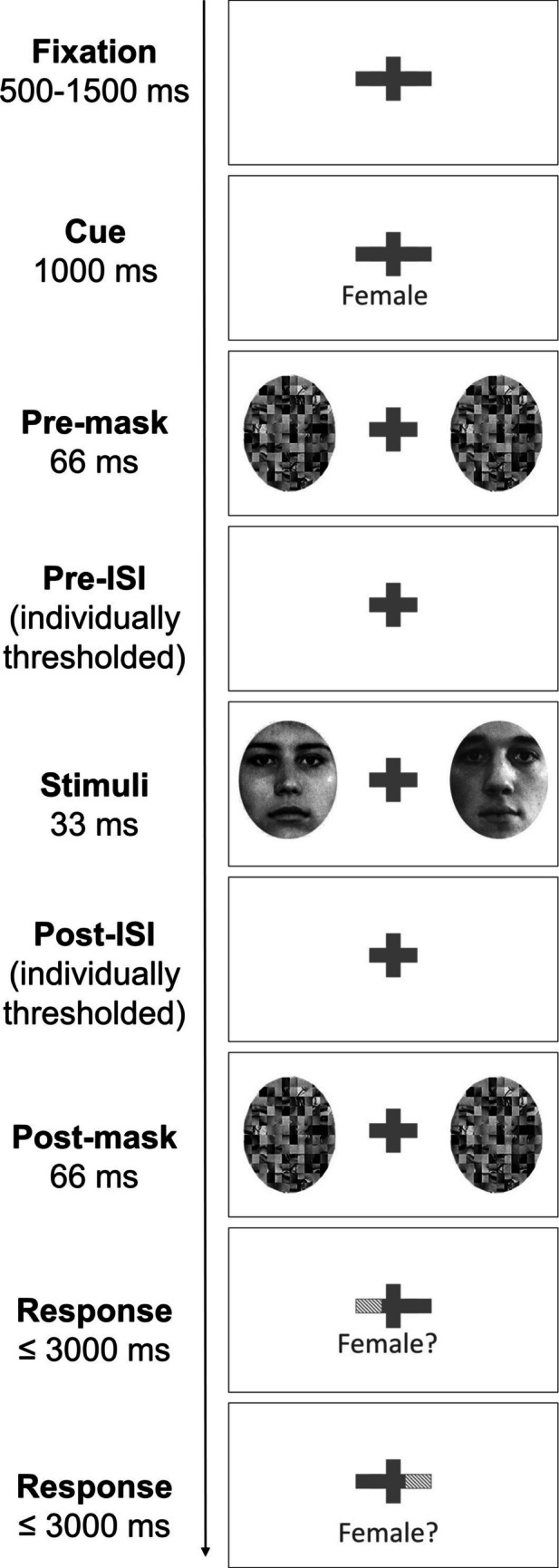
Dual-task trial sequence example (sex judgment trial, LVF response probed first). All trials began with a fixation period (duration varied randomly between 500 and 1,500 ms with 1-ms temporal resolution), followed by a 1,000-ms cue period (with no cue given during uncued trials), a 66-ms bilateral scrambled-face mask, an individually thresholded prestimulus interstimulus interval (ISI), a 33-ms face pair, a second ISI of the same duration as the first, another 66-ms bilateral scrambled-face mask, and two sequential end-of-trial response periods (response-terminated; maximum 3000 ms). During the response periods, text informing participants of the button mapping appeared above the fixation cross (not shown). Cued (single-task) trials only differed by the presence of a cue (cyan bar; depicted here with grey stripes) to either the LVF or RVF during the 1,000-ms cue period and collection of only one response for the cued side during the response collection period. Note that stimuli and masks are not shown to scale

Participants first completed a practice session in which they performed five trials for each of the six *judgment* categories to familiarize themselves with the general pacing of the experiment and the button mapping for entering responses. After completing the practice, participants completed three separate adaptive staircase procedures, one for each *judgment* type, to obtain approximately equivalent percentage-correct accuracy (around 75%, as in White et al., 2020) across the different *judgment* types. Participants first completed a staircase for the sex judgment to obtain an inter-stimulus interval (ISI) between the presentation of the pre- and postmasks and the face stimuli (see Fig. 2). The obtained ISI was then held constant for the remaining two staircase procedures (and the main experiment). In random order, participants completed a staircase on the orientation judgment (in which face visibility was manipulated by thresholding the transparency of an opaque Gaussian noise mask) and the color judgment (in which the visibility of the red tint was manipulated by thresholding the transparency of a red-tinted mask). The average ISI was 110 ± 7 ms. The mean alpha value for the orientation judgment stimuli was 0.69 ± 0.009 and the mean alpha value for the color judgment stimuli was 0.07 ± 0.002 (alpha ranges from 0, fully transparent, to 1, fully opaque). See the Supplemental Materials, S.4., for further details.

For the main experiment, participants completed 480 trials during a single session. The main experiment was divided into 24 blocks of 20 trials each. Each type of *cue* (single- or dual-task) was individually blocked; LVF and RVF cued trials were interleaved within each single-task block. Participants completed eight blocks (four single-task and four dual-task) for each *judgment* type with a self-timed break between each block. The blocks for each *judgment* type were divided such that participants completed four blocks (two single-task and two dual-task) of each intended target category per *judgment* (female or male, upright or inverted, and red-tinted or greyscale). Each *judgment* category was grouped so that participants completed the entirety of one category before experiencing the second *judgment* category; for example, observers completed all blocks asking whether the faces belonged to the “female” category before starting blocks asking whether the faces belonged to the “male” category. As in White et al. (2020), who adjusted threshold values throughout their main experiment, we too adjusted thresholds throughout: if accuracy was too high (>90%) or too low (<70%) after completing the four blocks for one *judgment* category, the respective threshold (ISI or alpha value) was adjusted for the next four blocks of the opposite *judgment* category to maintain approximately 80% mean accuracy for the *judgment* type. Block and trial orders were pseudorandomized for each participant.

### Trial sequence

A trial sequence example is shown in Fig. 2. At the start of each trial, a fixation cross flanked by a cue bar on either side appeared at the center of the screen for 500–1,500 ms (jittered in 1-ms intervals to encourage maintenance of vigilance between trials). In the ensuing cue period, both cue bars remained grey in the dual-task condition (as in Fig. 2), whereas one of the two cue bars changed to cyan in the single-task condition, and observers were reminded of *judgment* type via on-screen text. After this 1,000-ms cue period, dynamic premasks (described earlier) appeared centered 3.2° to the left and the right of the fixation cross for 66 ms. After a brief prestimulus interstimulus interval (ISI), a pair of face stimuli appeared in the same locations for 33 ms. Finally, after a poststimulus ISI period of the same duration as the first, a pair of dynamic postmasks appeared in the same locations for 66 ms. During the trial sequence, fixation breaks were monitored by recording the binocular gaze position of each participant using a GP3 eye-tracker system with a 60 Hz refresh rate (Gazepoint, Vancouver, BC). If the participant’s gaze fell beyond 2.4° horizontally from the fixation cross, the participant was presented with a warning (“Gaze moved too far”) on the screen, and the trial sequence was restarted from the fixation period (see Supplemental Materials, S.5., for details).

After the trial sequence concluded, participants were presented with the response screen(s). As in White et al. (2018) and Popovkina et al. (2021), participants were asked to indicate whether or not the stimulus belonged to the given category (“yes” or “no”) along with their level of confidence (“sure” or “guess”). A cyan cue bar would appear indicating to which side the participant was meant to respond. In the single-task trials, the cue bar always matched the initial cue at the start of the trial. In the dual-task trials, judgments were made on both items consecutively, with the cues appearing in a random order and a 100-ms delay between responses. The presented stimulus in each location had a 50% chance of belonging to the given category. This probability was also independent of the visual field, such that the correct response to one face did not reveal anything about the correct response to the second face (in dual-task trials). Participants used their left hand to respond to the face in the LVF or their right hand to respond to the face in the RVF. With their left hand, participants selected one of the following four keys: “f” (yes, confident), “d” (yes, guess), “s” (no, guess), or “a” (no, confident). With their right hand, the mapping was mirrored, such that participants selected one of the following four keys: “h” (yes, confident), “j” (yes, guess), “k” (no, guess), or “l” (no, confident). The mirroring was intended to aid participants, such that the same finger on each hand corresponded to the same response in each respective visual field. The appropriate button mapping appeared above the fixation cross during each response as a reminder to the participant as to which keys could be used to respond. Observers were given feedback after responding on each trial (the fixation cross flashed green, indicating correct responses, or red, indicating incorrect responses); if no response was entered after 3000 ms the trial was terminated and the next trial began.

## Results

Performance in each condition of the main experiment was quantified as A_g_, computed as the area under the receiver operating characteristic (ROC) curve (see Supplemental Fig. S2). A_g_ is a bias-free measure of accuracy that uses proportions of hits and false alarms (along with confidence data) to compute accuracy while accounting for response bias (e.g., an observer being more like to say “no” about category belonging than “yes”; Pollack & Hsieh, 1969). Like proportion correct, A_g_ is bounded by 0.5 (chance) and 1.0 (ceiling). See Supplemental Materials, S.6., for further details. Prior to conducting our main statistical analyses, we excluded 25 participants based on inadequate eye-tracking data (see Supplemental Materials, S.5., for details) and four participants for having accuracy at ceiling (A_g_ = 1.0) or below chance (A_g_ < 0.5) in the left visual field (LVF) or right visual field (RVF) of the single-task *cue* condition, resulting in a final set of 47 participants for analysis. Unless otherwise stated, we report descriptive statistics as the mean plus or minus standard error of the mean (*SEM*).

**Fig. 3:**
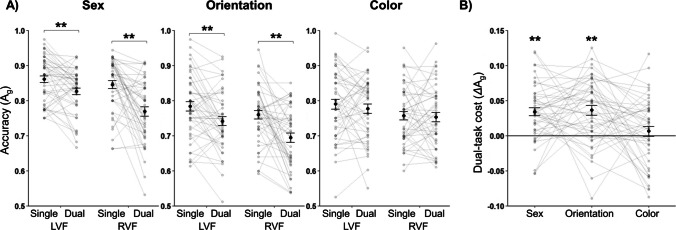
Accuracy (A_g_) and dual-task cost (*Δ*A_g_) results for all three *judgment* types. Black points represent the group-level mean A_g_ (±*SEM*) in (A) or mean *Δ*A_g_ (± SEM) in (B), and grey points (connected by grey lines) represent individual participant results. **A** Mean A_g_ for each *judgment* type for each *cue × location* condition. For face sex and orientation (i.e., face-based) judgments, but not judgments of color, mean A_g_ was higher in the single-task condition than in the dual-task condition (**p < .01 for paired-samples *t* tests). **B** Mean *Δ*A_g_ for each *judgment* type; larger *Δ*A_g_ values indicate greater dual-task cost. Judgments of face sex and orientation (but not color) showed significant dual-task costs (*Δ*A_g_ > 0; **p < .01 for one-sample *t* tests)

As shown in Fig. 3A, mean dual-task A_g_ was never higher than the corresponding mean single-task A_g_, and mean A_g_ was always higher for the LVF versus RVF (matched by *cue* condition, i.e., either single-task or dual-task). Mean A_g_ varied across the three *judgment* types. To assess the statistical significance of the effects of each of our conditions on A_g_, we conducted separate 2 × 2 repeated-measures analyses of variance (ANOVAs) for each *judgment* type: *cue* (single-task, dual-task) × *location* (LVF, RVF). The results of these ANOVAs confirmed statistically significant effects of *cue* on A_g_ for judgments of sex and orientation (but not for color), significant hemifield *location* asymmetries for all three *judgment* types (LVF A_g_ > RVF A_g_), and a significant *cue* × *location* interaction for judgments of face sex (but not color or orientation), such that LVF A_g_ > RVF A_g_ only occurred in the dual-task condition. On average, response time was faster for single-task as compared with dual-task trials for all three *judgment* types (Table S1). Finally, we found no effect of dual-task probe order and we therefore used mean dual-task A_g_ (averaged over dual-task Response 1 and Response 2) for all analyses. See Supplemental Materials, S.7., for the details of these preliminary analyses.

### Dual-task costs

We calculated a *Δ*A_g_ index for the dual-task cost as the difference between mean single- and dual-task A_g_ divided by the sum of these values to allow direct comparison of normalized dual-task costs between the different *judgment* types. Figure 3B shows mean *Δ*A_g_ for all three *judgment* types. We observed dual-task costs of similar magnitude for judgments of face sex and orientation, but no such cost for color judgments. Bonferroni-corrected one-sample *t* tests showed that *Δ*A_g_ > 0 for both sex (*M* = 0.03 ± 0.01), *t*(46) = 5.80, *p* < .001, *d* = 0.85, and orientation (*M* = 0.04 ± 0.01), *t*(46) = 5.35, *p* < .001, *d* = 0.78, but not color judgments (M = 0.01 ± 0.01), *t*(46) = 0.92, *p* = 1.00, *d* = 0.14. Using three Bonferroni-corrected paired-samples *t* tests, we found that color judgments differed from judgments of both face sex, *t*(46) = 3.29, *p* = .006, *d* = 0.48, and orientation, *t*(46) = 3.00, *p* = .013, *d* = 0.44, but that there was no discernable difference between the face sex and orientation judgments, *t*(46) = −0.22, *p* = 1.00, *d* = −0.03.

### Attention operating characteristic (AOC)

**Fig. 4:**
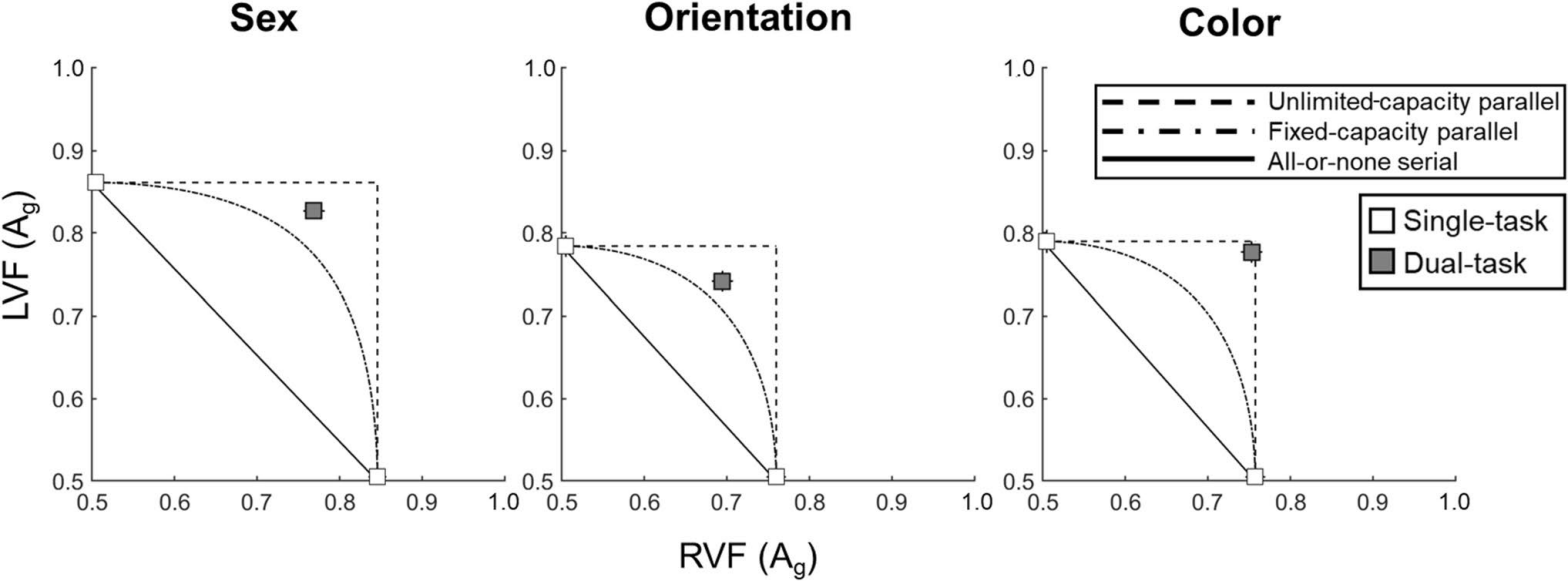
Attention operating characteristics (AOC). AOCs show the dual-task cost as dual-task performance (grey squares) relative to the single-task performance (unfilled squares pinned to axes) for the sex judgment (left), orientation judgment (middle), and color judgment (right). The results for the color judgment were best fit by the unlimited-capacity parallel model (intersecting dashed lines). For both the sex and orientation judgments, the results showed a larger cost than would be expected by the unlimited-capacity parallel model and instead showed a modest cost best fit by the fixed-capacity parallel model (dot-dashed curve). There was no evidence of all-or-none serial processing (solid diagonal line). Error bars represent standard error of the mean

We next assessed dual-task (versus single-task) costs with respect to three benchmark models of processing capacity used in previously published studies with a similar experimental method (Campbell et al., 2024; Popovkina et al., 2021, 2023; White et al., 2018, 2020):

1. An unlimited-capacity parallel model, which assumes that two items can be processed simultaneously without incurring a dual-task cost (intersecting dashed lines, Fig. 4);
2. A fixed-capacity parallel model, which assumes that processing resources are fixed and must be shared across the visual scene, resulting in a moderate cost (dash-dot curve; Fig. 4; Palmer, 1990; Scharff et al., 2011); and
3. An all-or-none serial model (solid diagonal line; Fig. 4), which assumes that only one of the two items can be fully processed at a time with no time to switch and process the second item, incurring a large dual-task cost.

To decide which benchmark model best fit the dual-task cost for each *judgment* type, we plotted single- and dual-task performance on attention operating characteristic plots (AOC; Sperling & Melchner, 1978). Figure 4 shows the AOC plots for the sex (left panel), orientation (middle panel), and color (right panel) judgments. In all three plots, the x-axis represents mean A_g_ on the RVF cued trials, and the *y*-axis represents mean A_g_ on the LVF cued trials. Mean dual-task A_g_ for each side is plotted within the space of the plot. To test which model best fit the reported costs, we assessed the mean distance of the dual-task point to the models of interest. As others have reported (Campbell et al., 2024; Popovkina et al., 2021, 2023; White et al., 2018, 2020), we calculated the shortest Euclidean distance to the all-or-none serial diagonal and to the fixed-capacity parallel curve for each observer. Distances were assigned a negative value if they fell below (i.e., to the left of) the model of interest and a positive value if they fell above the model of interest. We report one-sample *t* tests comparing the mean distances to a distance of 0.

#### Sex

As shown in Fig. 4 (left panel), the results of the sex judgment fell intermediate to the fixed-capacity parallel model and the unlimited-capacity parallel model. The mean distance from the all-or-none serial line was 0.17 ± 0.01, which was significantly larger than 0, *t*(46) = 14.16, *p*_*Bonferroni*_ < .001, *d* = 2.06. In addition, the distance from the fixed-capacity curve was 0.05 ± 0.01, which was also larger than 0, *t*(46) = 4.07, *p*_*Bonferroni*_ < .001, *d* = 0.59. The magnitude of the cost (*M* = 0.06 ± 0.01) was larger than predicted by the unlimited-capacity parallel model, *t*(46) = 5.90, *p* < .001, *d* = 0.86. We take this as evidence that the results for the sex judgment were best predicted by the fixed-capacity parallel model.

#### Orientation

As shown in Fig. 4 (middle panel), the results of the orientation judgment were also intermediate to the fixed-capacity parallel model and the unlimited-capacity parallel model. The mean distance from the all-or-none serial line was 0.12 ± 0.01, which was significantly larger than 0, *t*(46) = 9.69, *p*_*Bonferroni*_ < .001, *d* = 1.41. The distance from the fixed-capacity curve was 0.04 ± 0.01, which was also significantly greater than 0, *t*(46) = 3.14, *p*_*Bonferroni*_ = .006, *d* = 0.46. Despite mean dual-task A_g_ falling above the fixed-capacity parallel curve, the magnitude of the cost was larger than predicted by the unlimited-capacity parallel model (*M* = 0.05 ± 0.01), *t*(46) = 5.40, *p* < .001, *d* = 0.79. Thus, the results for orientation categorization were best predicted by the fixed-capacity parallel model.

#### Color

Finally, as shown in Fig. 4 (right panel), the distance from both the all-or-none serial model (*M* = 0.18 ± 0.01), *t*(46) = 13.08, *p*_*Bonferroni*_ < .001, *d* = 1.91, and the fixed-capacity parallel model (*M* = 0.10 ± 0.01), *t*(46) = 7.08, *p*_*Bonferroni*_ < .001, *d* = 1.03, were greater than 0. As reported in the ANOVA earlier, we observed no evidence of a dual-task cost (M = 0.01 ± 0.01), *t*(46) = 0.82, *p* = .417, *d* = 0.12. Thus, the results of the color judgment were best fit by the unlimited-capacity parallel model.

#### Congruency

**Fig. 5:**
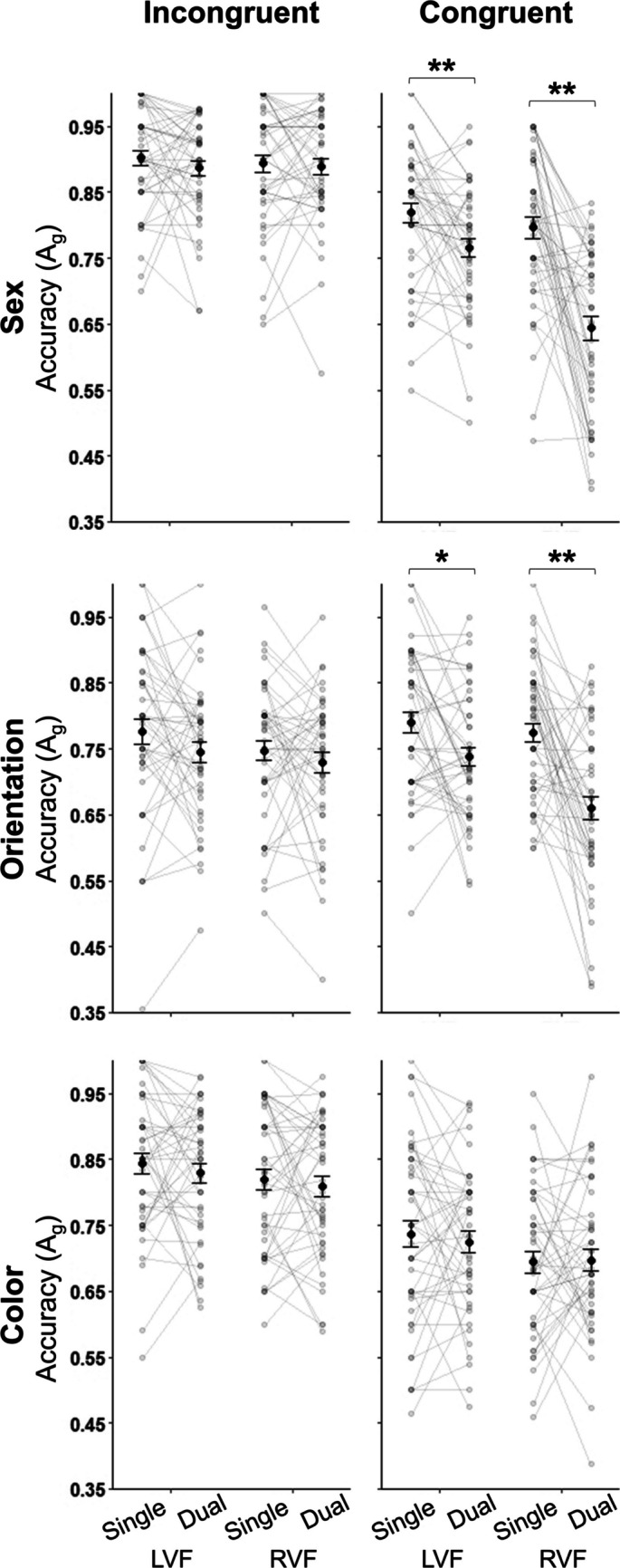
Accuracy (A_g_) results for all three *judgment* types split by congruency (incongruent, left column; congruent, right column). Black points represent the mean A_g_ (±*SEM*) at the group level, and grey points (connected by grey lines) represent individual participant results. For all three *judgment* types, single-task A_g_ did not differ from dual-task A_g_ when stimuli were category-incongruent. For judgments of face sex and orientation (i.e., face-based judgments), but not judgments of color, mean A_g_ was higher in the single-task condition than in the dual-task condition when face stimuli were category-congruent. Statistically significant differences indicated by asterisks for *p < .05, **p < .01 in paired comparisons (asterisks placed above horizontal bars). Error bars represent standard error of the mean

As an additional test of capacity, the studies by White and colleagues (Campbell et al., 2024; White et al., 2018, 2020) reported effects of category congruency for their stimulus pairs. Pairs of stimuli in which both faces belonged to the same judgment-relevant category (e.g., female–female, upright–upright, tinted–tinted) were considered congruent, while pairs of stimuli in which each face belonged to a different judgment-relevant category (e.g., female–male, upright–inverted, red tinted–greyscale) were considered incongruent. Figure 5 shows A_g_ for the different types of category-congruency for each *cue* and *location* condition in each *judgment* type (corresponding statistical analyses are included in the Supplemental Materials, S.8.). For judgments of face sex and color, we found an incongruency advantage, such that mean A_g_ was greater for incongruent stimuli than for congruent stimuli (no corresponding differences were observed in response time; see Table S2). No such incongruency advantage was found for the orientation judgment. An LVF advantage emerged along with dual-task costs for the sex and orientation judgments when stimuli were congruent, but no such hemifield asymmetries or costs were observed when stimuli were incongruent. Finally, the color judgment showed no evidence of dual-task costs or hemifield asymmetries for either incongruent or congruent stimuli. We determined the statistical significance of these observations using separate 2 × 2 × 2 repeated-measures ANOVAs for each *judgment* type: *congruency* (incongruent, congruent) × *cue* (single, dual) × *location* (LVF, RVF). See Supplemental Materials, S.8, for the details of these analyses. In addition to analyzing A_g_ differences related to congruency, we also calculated a *Δ*A_g cong_ index of congruency cost, calculated as the difference between mean congruent and incongruent A_g_ divided by the sum of these values for both the single-task and the dual-task conditions in each *judgment* type. For both of the face-based judgments, we observed greater effects of congruency in the dual-task relative to the single-task; this was not the case for the color judgments, which showed equivalent congruency effects regardless of *cue* condition (see Supplemental Materials, S.8.3).

### LVF advantage

**Fig. 6:**
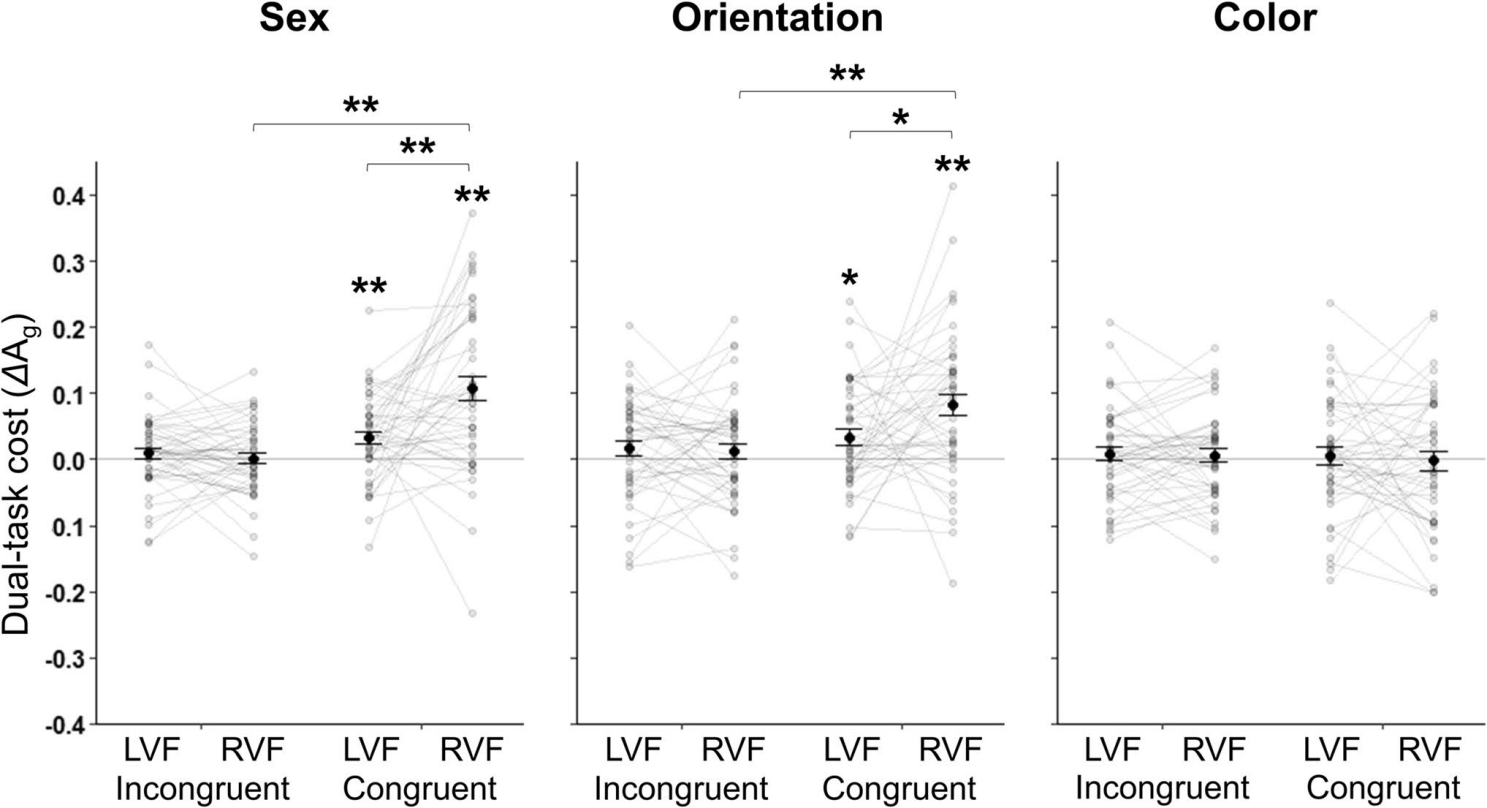
Effect of stimulus congruency and *cue* on dual-task cost and hemifield cost asymmetry (as in Fig. 3, larger *Δ*A_g_ values indicate decreased dual-task A_g_ relative to single-task A_g_). Black points represent the mean *Δ*A_g_ (±*SEM*) at the group level, and grey points (connected by grey lines) represent individual participant results. None of the three *judgment* types showed dual-task costs or hemifield cost asymmetries for incongruent trials. For congruent trials, judgments of face sex and orientation (but not color) showed dual-task costs (i.e., *Δ*A_g_ > 0) for both LVF and RVF judgments. Pairwise statistical tests on LVF–RVF dual-task cost differences for congruent trials showed a cost asymmetry for face sex and orientation judgments (but not for color judgments). Additional pairwise comparisons showed greater congruent-trial versus incongruent-trial costs in the RVF (but not the LVF) for both face sex and orientation judgments. Statistically significant differences indicated by asterisks for *p < .05, **p < .01 for both one-sample *t* tests (asterisks directly above individual data points) and for one-tailed, paired-samples *t* tests (asterisks above horizontal bars). Error bars represent standard error of the mean

Finally, we assessed the effect of *cue* and congruency on hemifield asymmetry by examining the difference between mean A_g_ in LVF and RVF judgments for incongruent and congruent trials. For each type of *judgment*, we calculated another *Δ*A_g_ index for dual-task cost as the difference between mean single- and dual-task A_g_ divided by the sum of these values at each *location* (LVF/RVF) for each type of category-congruency (incongruent/congruent). Figure 6 shows mean *Δ*A_g_ for each type of category congruency at each *location* across the three *judgment* types. We then conducted a 3 (*judgment*: sex, color, orientation) × 2 (*congruency*: incongruent, congruent) × 2 (*location*: LVF, RVF) repeated-measures ANOVA comparing the normalized *Δ*A_g_ index values. Since the three-way interaction was significant, *F*(2, 92) = 4.98, *p* = .009, η_p_^2^ = 0.10, we next conducted a 2 (*congruency*: incongruent, congruent) × 2 (*location*: LVF, RVF) repeated-measures ANOVA at each level of *judgment*.

#### Sex

As shown in Fig. 6 (left panel), dual-task costs for the sex judgments were largely driven by a cost in the RVF when stimuli were congruent. We observed significant main effects of *congruency*, *F*(1, 46) = 32.44, *p* < .001, η_p_^2^ = 0.41, and *location*, *F*(1, 46) = 8.63, *p* = .005, η_p_^2^ = 0.16. The effect of *location* depended on stimulus *congruency*, as evidenced by a two-way interaction, *F*(1, 46) = 14.28, *p* < .001, η_p_^2^ = 0.24.

Of primary interest was a prospective LVF advantage in dual-task cost. To test for this possibility, we next conducted four planned one-tailed, paired-samples *t* tests with Bonferroni correction. The first pair of tests examined LVF–RVF differences within both incongruent and congruent trials. While no difference was observed between the LVF (*M* = 0.01 ± 0.01) and RVF (*M* = 0.002 ± 0.01) for incongruent trials, *t*(46) = −0.72, *p* = .956, *d* = −0.10, a difference was apparent for congruent trials such that the dual-task cost in the RVF (*M* = 0.11 ± 0.02) was greater than in the LVF (*M* = 0.03 ± 0.01), *t*(46) = 3.71, *p* = .001, *d* = 0.54. The second pair of *t* tests determined whether costs differed between types of category-congruency by comparing costs between incongruent and congruent trials at the same *location* (LVF-LVF and RVF-RVF). Dual-task costs did not differ between incongruent and congruent trials in the LVF, *t*(46) = 1.91, *p* = .124, *d* = 0.28, but the dual-task cost was greater for congruent trials relative to incongruent trials in the RVF, *t*(46) = 5.73, *p* < .001, *d* = 0.84.

Bonferroni-correct two-tailed, one-sample *t* tests used to determine whether *Δ*A_g_ > 0 in each hemifield revealed significant dual-task costs in both the LVF, *t*(46) = 3.42, *p* = .005, *d* = 0.50, and RVF, *t*(46) = 5.98, *p* < .001, *d* = 0.87, for congruent trials, but no such cost for incongruent trials, LVF: *t*(46) = 1.07, *p* = 1.00, *d* = 0.16; RVF: *t*(46) = 0.24, *p* = 1.00, *d* = 0.04.

#### Orientation

As in the sex judgment results, dual-task costs for the orientation judgments were driven by a cost for congruent stimuli in the RVF, as shown in Fig. 6 (middle panel). A *congruency* × *location* ANOVA for the orientation judgment revealed a main effect of *congruency, F*(1, 46) = 11.42, *p* = .001, η_p_^2^ = 0.20, but not of *location, F*(1, 46) = 2.76, *p* = .104, η_p_^2^ = 0.06. The ANOVA also showed an interaction between *congruency* and *location, F*(1, 46) = 4.81, *p* = .033, η_p_^2^ = 0.10.

Using the same planned one-tailed, paired-samples *t*-tests as described earlier, we found no difference between LVF (*M* = 0.02 ± 0.01) and RVF (*M* = 0.01 ± 0.01) *Δ*A_g_ values for incongruent trials, *t*(46) = −0.32, *p* = 1.00, *d* = −0.05, but found an LVF cost advantage for congruent trials (LVF: *M* = 0.03 ± 0.01; RVF: *M* = 0.08 ± 0.02), *t*(46) = 2.35, *p* = .047, *d* = 0.34. While dual-task costs did not differ between incongruent and congruent trials in the LVF, *t*(46) = 0.91, *p* = .738, *d* = 0.13, the cost was larger for congruent trials relative to incongruent trials in the RVF, *t*(46) = 3.92, *p* < .001, *d* = 0.57.

Bonferroni-correct two-tailed, one-sample tests revealed *Δ*A_g_ > 0 in the LVF, *t*(46) = 2.88, *p* = .024, *d* = 0.42, and RVF, *t*(46) = 4.95, *p* < .001, *d* = 0.72, for congruent trials, but no such dual-task costs for incongruent trials, LVF: *t*(46) = 1.54, *p* = .519, *d* = 0.22; RVF: *t*(46) = 1.09, *p* = 1.00, *d* = 0.16.

#### Color

In contrast to the last two ANOVAs, the *congruency* × *location* ANOVA for color judgments failed to show a main effect of *congruency, F*(1, 46) = 0.17, *p* = .685, η_p_^2^ = 0.004, or *location. F*(1, 46) = 0.18, *p* = .672, η_p_^2^ = 0.004, on the cost, and showed no evidence of a *congruency* × *location* interaction, *F*(1, 46) = 0.07, *p* = .795, η_p_^2^ = 0.001. As shown in Fig. 6 (right panel), dual-task costs were not observed for either incongruent or congruent trials, regardless of hemifield.

Using the same planned one-tailed, paired-samples *t* tests as described earlier, LVF (incongruent: *M* = 0.01 ± 0.01; congruent: *M* = 0.01 ± 0.01) and RVF (incongruent: *M* = 0.01 ± 0.01; congruent: *M* = −0.002 ± 0.01) costs did not differ for incongruent trials, *t*(46) = −0.20, *p* = 1.00, *d* = −0.03, or for congruent trials, *t*(46) = −0.42, *p* = 1.00, *d* = −0.06. Finally, no difference was observed between incongruent and congruent trials in the LVF, *t*(46) = −0.18, *p* = 1.00, *d* = −0.03, or in the RVF, *t*(46) = −0.46, *p* = 1.00, *d* = −0.07.

Using Bonferroni-correct two-tailed, one-sample *t*-tests to compare the magnitude of each cost to 0 revealed no strong cost for any condition (all corrected *p* values = 1.00 and all *d* values < 0.20). The effect of *congruency* equivalently reduced mean A_g_ across each *cue* and *location* condition when stimuli were congruent without incurring a dual-task cost.

### Controlling for inflated sex judgment accuracy

Accuracy (A_g_) was somewhat higher for the sex judgment than for the orientation and color judgments, particularly for incongruent trials (Figs. 3 and 5). To determine whether elevated A_g_ had an effect on lateralized dual-task costs, we performed a final set of analyses—consistent with those already reported—on a subset of the sex judgment data (obtained by imposing a strict A_g_ cutoff for incongruent trials, resulting in participants being removed from analysis if A_g_ was too high) supplemented with data from additional participants acquired to achieve sufficient statistical power (see Supplemental Materials, S.9., for details). Despite a reduction in overall accuracy (from A_g_ > 0.8 to A_g_ < 0.8), the accuracy-controlled sex judgment results still showed a dual-task cost and greater A_g_ in the LVF than in the RVF (Fig. S4A, as compared with Fig. 3A). Additionally, while the dual-task cost index (*Δ*A_g_) increased slightly, the magnitude of this cost index was still not consistent with magnitudes reported in previously published studies claiming severely limited processing capacity (e.g., Popovkina et al., 2021; White et al., 2018, 2020; Fig. S4B, as compared with Fig. 3B). Furthermore, these results were better fit by a fixed-capacity parallel model than an all-or-none serial model (Fig. S4C). With regard to A_g_ split by congruency, we again found no dual-task costs or LVF–RVF differences when stimuli were incongruent and comparable dual-task costs and LVF–RVF differences when stimuli were congruent (compare Fig. S5A to the corresponding two top panels of Fig. 5). When accuracy was controlled, congruency costs (*Δ*A_g cong_) were reduced to 0 in the single-task condition (Fig. S5B; unlike Fig. S3), but were still apparent in the dual-task condition (compare Fig. S5B to Fig. S3). Notably, lateralized dual-task costs—the result of primary interest—were still apparent for congruent trials (compare Fig. S6 to the left panel of Fig. 6), demonstrating the generalizability of the LVF cost advantage. See Supplemental Materials, S.9., for the details of these analyses.

## Discussion

We used a dual-task paradigm to study the interaction of lateralized face perception and attention. We were particularly interested in determining whether visual field asymmetries in face perception would be related to divided attention costs in the visual processing of faces viewed in opposite hemifields. We adapted the logic and general method of recent studies that showed divided attention costs for the visual processing of non-face stimuli (Campbell et al., 2024; Popovkina et al., 2021, 2023; White et al., 2018, 2020). Our paradigm required observers to perform binary perceptual judgments for either one (single-task) or both (dual-task) of two simultaneously viewed faces, which allowed us to measure the potential costs of dividing attention as a decrease in accuracy for dual-task versus single-task judgments. As in some of the studies just mentioned (White et al., 2018, 2020), we hypothesized that costs would be observed unless a judgment could be performed on the basis of basic visual feature (i.e., color) information. We were particularly interested in the possibility of lateralized dual-task costs for face sex and orientation (i.e., “face-based”) judgments, but not for color judgments that could be performed without cost and without perceiving the face per se. We reasoned that dual-task costs could be accompanied by a left visual field (LVF) advantage depending on the degree to which a given judgment engages right-lateralized face processing mechanisms that process face information better in the LVF than in the right visual field (RVF).

We chose judgments of sex and orientation because these face-based judgments can be performed relatively easily and accurately (e.g., Bruce et al., 1993) with respect to binary categories, but nevertheless differ with respect to the relevance of social trait information. Judgments of face orientation can be made without reference to social trait information and in this sense are potentially more “shallow” (Bindemann et al., 2005) than judgments of face sex, and might therefore show less if any cost. Despite these potential differences, we observed similar dual-task costs and commensurate LVF advantages for the two judgments. In short, the costs of dividing attention for both face-based (but not color) judgments were largely driven by performance costs in the right visual field. Our results are consistent with known visual field asymmetries associated with the LVF advantage for face processing (e.g., Leehey et al., 1978; Rhodes, 1985; Sergent & Bindra, 1981; Yovel et al., 2003), and the possibility that when basic visual feature information is insufficient to make perceptual judgments of two faces in parallel, LVF face information is prioritized (Harrison & Strother, 2020) and subject to capacity-limited visual processing. Our results provide novel evidence of a direct relationship between lateralized face perception and dual-task costs of divided attention and thus contribute to scientific knowledge concerning the interaction of face perception and attention, which is relatively understudied as compared with each topic considered separately.

### Dual-task costs for face-based judgments

Effects of divided attention were limited to the sex and orientation judgments, and the dual-task costs observed for these judgments were of similar magnitude. The lack of dual-task costs for color judgments was expected based on the results of previous studies that used a similar method for non-face stimuli (White et al., 2018, 2020; but as reported by Fitousi, 2019, other paradigms can show different capacity-related results). The feature-based color judgments provided a crucial processing baseline against which to compare results for the face-based judgments because, unlike judgments of face sex and orientation, judgments of face color did not require visual processing of information conveyed by the underlying face upon which a red tint was superimposed. This suggests that the costs observed for the judgments of face sex and orientation are related to the type of visual processing common to these face-based judgments and that even small costs might reflect an insufficiency of basic visual feature information to perform the task.

Our choice of face sex judgments was motivated by previous studies that reported serial bottlenecks for the perception of face sex (Bindemann et al., 2005). We wondered if such a severe capacity limit would generalize to our paradigm, which was designed specifically to test for serial bottlenecks related to perceptual and semantic processing using a model-based approach (Campbell et al., 2024; Popovkina et al., 2021, 2023; White et al., 2018, 2020). Although we observed a clear cost of divided attention for judgments of face sex, model-based analyses based on the magnitude of this cost did not favor an all-or-none serial processing account of our results. In fact, the dual-task costs reported here for faces were surprisingly small—substantially smaller than those reported in the studies just mentioned (i.e., at most 63% of the cost reported by Popovkina et al., 2021, whose costs were intermediate to their all-or-none serial and fixed-capacity parallel models; for direct comparison of cost magnitudes, see Fig. S4B). This also applied to judgments of face orientation (which also showed no compelling evidence of all-or-none serial limitations in our model-based analyses), the use of which in our experiment was motivated by conflicting results concerning the existence of a bottleneck for face detection (Fitousi, 2021; Qarooni et al., 2022). Both face detection and the perception of face orientation are associated with first-order configural information (Maurer et al., 2002; Piepers & Robbins, 2012), which could show a capacity limit but not necessarily an all-or-none serial bottleneck. In a later section of our discussion, we will return to the possibility of serial processing for certain kinds of face perception as it relates to the magnitude of our costs as compared with those reported by Popovkina et al. (2021, 2023) and White et al. (2018, 2020) for non-face stimuli.

As mentioned earlier, we observed dual-task costs for both of our face-based judgments, which were of similar magnitude for each judgment type (despite physical differences between the stimuli for each). We were surprised by this because the two judgments, while both face-based and rudimentary, differ qualitatively. Unlike judgments of face sex, judgments of face orientation do not require the perception of social trait information. Additionally, whereas first-order configural information is sufficient for judgments of face orientation (Maurer et al., 2002; Piepers & Robbins, 2012), it is not necessarily sufficient to perform a judgment of face sex—which is made on the basis of complex combinations of local and configural information (Baudouin & Humphreys, 2006; Brown & Perrett, 1993; Bruce et al., 1993; Schyns et al., 2002; Yamaguchi et al., 2013; Yokoyama et al., 2014; Zhao & Hayward, 2010). Thus, our failure to observe differences in the magnitude of the costs for the two judgment types might reflect either (1) a common bottleneck at an early stage of face processing that limits the amount of face information that can be processed on the basis of first-order configural information, with little if any additional cost incurred by perceptual (but not postperceptual; Harrison et al., 2022) processing beyond this stage, or (2) the coincidental occurrence of costs of similar magnitude that result from capacity limits associated with distinct mechanisms and processes involved in each judgment type. Regardless, we conclude that the costs noted here appear to reflect the face-based nature of face sex and orientation judgments rather than differences in visual processing required to perform each type of face-based judgment (i.e., because we did not see differences in cost between the two judgment/stimulus types). We return to this later when we discuss LVF–RVF dual-task cost asymmetries. Due to the different stimulus manipulations used for each judgment type, we note the need to use caution when interpreting direct comparisons between judgments. Additionally, although we reported response time data in the Supplemental Materials (S.7.3), we found that response time differences—which occurred for all three judgment types—did not reflect differences in cost (*Δ*A_g_) per se.

### Feature contrast

An unexpected but informative aspect of our results emerged from statistical tests of “congruency” effects (using a similar approach to Campbell et al., 2024; White et al., 2018, 2020), which often result in better performance for congruent stimuli relative to incongruent stimuli. Previously published studies (Campbell et al., 2024; White et al., 2018, 2020) reported congruency effects in the expected direction (congruent > incongruent accuracy). These authors argued that congruency effects may emerge for a serial process when participants do not attend properly to the cued side or swap dual-task responses (i.e., “cross-talk”). As we will discuss, the difference in congruency effects between the results reported here and those of previously published studies may also be due at least in part to the type of stimuli used: face-based judgments can be made differently than word-based judgments, which contain no diagnostic visual cues.

In contrast to an expected interference effect (i.e., decreased accuracy) for category-incongruent face pairs, we observed an “incongruency benefit” for both color judgments and face sex judgments (but more limited effects of congruency for judgments of face orientation), such that overall accuracy was better for category-incongruent (e.g., female–male, tinted–greyscale) trials as compared with category-congruent trials (e.g., female–female, tinted–tinted). Although this was not expected for judgments of face sex, our finding of an incongruency benefit for color judgments is not unprecedented because it is consistent with the results of White et al. (2018), who used the same dual-task paradigm and a color judgment similar to ours (using red-tinted words rather than faces). We therefore adopt the explanation offered by White et al. (2018) to account for our color-incongruency advantage, which was that observers can compare color information (or lack thereof) between spatially disparate stimuli to determine whether color was present or not more accurately as compared with judging each stimulus independently (i.e., a *feature-contrast* comparison of feature information from both faces). We found that this occurred irrespective of cue: even when observers were cued to one face location, they apparently benefitted from color-based contrastive information to perform their judgments of color-incongruent face pairs.

The observation of a corresponding feature-contrast advantage for face sex judgments implies that, whichever cues observers used to judge the sex of faces, these cues provided contrastive information in addition to single-face information. As observed for dual-task color judgments, we observed higher dual-task accuracy for sex-incongruent versus sex-congruent face pairs (which was not accompanied by a corresponding increase in response time for either judgment type). The observation of this feature-contrast effect for judgments of face sex is interesting in its own right, and possibly related to the use of contrastive information to create distinctiveness between faces on the basis of complex visual features (Campanella et al., 2001; O’Toole et al., 1998; Rekow et al., 2020; Webster et al., 2004). We do not suspect that this effect was based on a contrast of basic visual feature information (e.g., mean luminance or color) or external face features (e.g., hair or face shape) sometimes associated with judgments of face sex (e.g., Bruce et al., 1993; González-Álvarez & Sos-Peña, 2022; Nestor & Tarr, 2008), because we controlled for these potential cues in our stimuli. Instead, we suspect that observers used other diagnostic internal face-feature information, such as second-order configural information about the spatial relations between local face features (Baudouin & Humphreys, 2006; Brown & Perrett, 1993). Of course, since we did not manipulate face-feature information we can only speculate.

Dual-task judgments of face sex showed a considerably stronger cost of congruency than did single-task judgments of face sex (even when accuracy was controlled; see Fig. S5B); this was also the case for orientation judgments but not for color judgments (see Fig. S3). Furthermore, when face pairs were congruent, dual-task costs were observed for both face-based judgments but not for color judgments (for which there were no dual-task costs, even for congruent trials; see Fig. 6). Across all three judgment types, dual-task costs were not observed when face pairs were incongruent. That is, like judgments of face sex, dual-task costs for judgments of face orientation were limited to judgments of category-congruent face pairs, though the difference in cost magnitude between the two judgment types could be due at least in part to stimulus differences. This means that, even though feature-contrast did not yield a benefit above and beyond single-task cueing for judgments of face orientation (or accuracy-controlled sex judgments; Fig. S5B), feature-contrast might have nevertheless precluded dual-task cost for orientation-incongruent face pairs, which suggests that feature-contrast can negate dual-task costs otherwise observed for our face-based judgment types. Taking into account the different ways stimulus congruency impacted accuracy and dual-task costs for the different judgment types, we conclude that interactions of congruency and divided attention reflect the negation of dual-task costs for face-based judgments of sex and orientation, but not feature-based color judgments.

### The LVF advantage

Throughout our analyses we observed hemifield asymmetries in both accuracy (A_g_) and dual-task cost (*Δ*A_g_). Consistent with a general LVF attentional bias (e.g., Benwell et al., 2014; Cicek, 2009; Heilman & Abell, 1980; Reuter-Lorenz et al., 1990), accuracy was greater in the LVF than in the RVF for all three judgment types, albeit greater for face-based judgments (of face sex and orientation) relative to feature-based (color) judgments. These analyses also showed that the LVF advantage in accuracy for judgments of face sex and orientation mostly reflected LVF–RVF accuracy asymmetries for dual-task judgments, specifically for congruent face pairs, which we explored further in our analyses of dual-task cost (*Δ*A_g_). While we observed a general LVF advantage for all three judgment types, an LVF advantage with respect to dual-task cost was restricted to our face-based judgments, which suggests that this LVF cost advantage is distinct from the general LVF bias in spatial attention. Dual-task costs were limited to congruent trials and were observed for both LVF and RVF faces. Notably, however, these costs were asymmetric between the LVF and RVF for both judgments of face sex and orientation.

Our observation that LVF–RVF asymmetries in the cost of dividing attention occurred for both face-based judgments is consistent with the possibility that capacity limits arise at an early stage of processing, and may relate to a reliance on holistic processing, even for inverted faces (e.g., Sun et al., 2025). The LVF cost advantage reported here might also be related to visual field asymmetries associated with a decreased reliance on feature-based processing (Cattaneo et al., 2014; Hillger & Koenig, 1991; Parkin & Williamson, 1987; Rhodes, 1985; Ross & Turkewitz, 1981; Rossion et al., 2000; Scott & Nelson, 2006; Sergent, 1982, 1985; Sergent & Bindra, 1981). The LVF–RVF asymmetries observed in our study demonstrate a clear relationship between lateralized face processing and divided attention: an LVF advantage that reflects asymmetries in both accuracy (LVF A_g_ > RVF A_g_) and cost (LVF *Δ*A_g_ < RVF *Δ*A_g_), and only occurs for faces belonging to the same judgment-specific category. This relationship was robust and generalized across differences in the stimuli used for each judgment type and corresponding differences in congruency effects. Based on the logic of divided field experiments (Bourne, 2006), our novel finding of an attention-contingent LVF advantage suggests a corresponding relationship between divided attention and right hemisphere superiority for holistic face processing (Harrison & Strother, 2020). While other studies have reported limits in the ability to perceptually process multiple faces in parallel (Bindemann et al., 2005, 2007; Brebner & Macrae, 2008; Fitousi, 2021; Jenkins et al., 2003; Olk & Garay-Vado, 2011; Palermo & Rhodes, 2002; Thoma, 2014; Thoma & Lavie, 2013), we are not aware of any other reports of a direct relationship between lateralized face processing and divided attention using a dual-task approach like the one used here—although other studies have combined attentional manipulations with hemifield-lateralized face stimuli (Klein, 1976; Liu et al., 2014; Moscovitch et al., 1976; Vuilleumier, 2000).

### Future directions

Our results are consistent with those of other studies indicating that capacity limits can occur during the earliest stages of face processing (e.g., Bindemann et al., 2005; Fitousi, 2021). It should be noted, however, that the magnitudes of the dual-task costs reported here are relatively small compared with previous studies that employed a nearly identical method to identify all-or-none serial bottlenecks for visual processing of non-face stimuli. Critically, however, these studies relied on semantic categorization judgments rather than the relatively “shallow” (i.e., not contingent upon identity-related semantic information; Bindemann et al., 2005) perceptual judgments performed in our experiment. Therefore, future studies could employ an identity-related semantic categorization task (e.g., categorization as a politician or pop star, as in Jenkins et al., 2003; Lavie et al., 2003; Thoma, 2014; Thoma & Lavie, 2013), but minimize cost confounds related to working memory and other postperceptual processes (Harrison et al., 2022), in order to make more direct comparisons of dual-task costs reported for non-face stimuli (Campbell et al., 2024; Popovkina et al., 2021, 2023; White et al., 2018, 2020). With respect to capacity and the hemifield cost asymmetries observed here, it is possible that the LVF cost advantage is related to the contralateral allocation of independent hemisphere-specific attentional resources (e.g., Alvarez & Cavanagh, 2005), and thus reflects both right hemisphere superiority and hemifield-hemisphere correspondence, but only in the absence of a cue. This could be investigated by presenting multiple faces viewed either between hemifields or within the LVF or RVF (Popovkina et al., 2023).

Given the relatively small costs reported here for face-based judgments, one interesting possibility is that our results could be explained using an interference account. For instance, it is possible that two faces could be perceived as well as a single face, but only when sufficiently dissimilar. Congruent faces (two faces that belong to the same category) might show more mutual interference due to visual similarity than two sufficiently dissimilar incongruent faces. In this case, costs might be better explained in terms of biased competition (Desimone & Duncan, 1995) as opposed to capacity limitations. Future studies could address this possibility and potentially elucidate the source of cost in terms of capacity limitations, biased competition, or both.

It is worth noting that the congruency effects observed in our study could, in principle, reflect participant strategy or response bias. For example, if an observer sees one face more clearly than another, that observer might be biased toward reporting that the second face belongs to the opposite category, which would result in better accuracy for incongruent relative to congruent trials. However, there is no clear reason to assume that participants would exhibit this type of bias over its opposite (choosing to report category congruency when the second face is less visible than the first). More importantly, such a bias alone cannot explain LVF–RVF cost asymmetry without the additional assumption of an LVF bias. Nevertheless, future studies could investigate this possibility further by disentangling a potential response bias from a hemifield bias. Future studies could also seek to explain the accuracy differences we observed for the different judgment types despite our use of an adaptive method to adjust difficulty for individual participants. Currently, we can only speculate that these might be related to differences in how stimulus visibility was thresholded for each judgment type: inter-stimulus interval for the sex judgments, noise mask transparency for orientation judgments, and red-tinted mask transparency for judgments of face color. It is also possible that the unexpected congruency effects contributed. Importantly, the LVF–RVF cost asymmetries observed for the sex judgments occurred even when controlling for elevated accuracy. Nevertheless, future studies could investigate ways to more effectively maintain similar accuracy across different judgment types.

## Conclusions

Dividing attention to two simultaneously viewed faces results in a dual-task cost for perceptual judgments that rely on face-based processing. This cost of dividing attention was surprisingly small and asymmetric between the LVF and RVF, such that dual-task, divided attention costs were greater for faces in the RVF than for faces in the LVF (i.e., an LVF cost advantage). Importantly, no costs of divided attention or corresponding LVF–RVF asymmetries were observed for simple feature-based (i.e., color) judgments of faces. While some previous studies have shown that single faces can be processed in the near absence of attention (Reddy et al., 2004, 2006), results from other studies suggest that there might be a limit to the number of faces that can be processed in parallel (Bindemann et al., 2005, 2007; Brebner & Macrae, 2008; Fitousi, 2021; Jenkins et al., 2003; Olk & Garay-Vado, 2011; Palermo & Rhodes, 2002; Thoma, 2014; Thoma & Lavie, 2013). Our study, however, is the first to show a relationship between the costs of dividing attention between two faces and LVF–RVF asymmetries associated with early stages of face processing. Our findings highlight the role of hemispheric asymmetries in face perception and the importance of considering laterality for a more complete understanding of how attention and face processing interact.

## Supplementary Information

Below is the link to the electronic supplementary material.

Supplementary file1 (DOCX 3557 KB)

## Data Availability

The data for this study are available on Open Science Framework (https://osf.io/gd93n). This experiment was not preregistered.

## Footnotes

1 As recommended (e.g., https://orwh.od.nih.gov/sex-gender), we use “sex” rather than “gender” to refer to judgments of face stimuli being perceived as either female or male (without a “nonbinary” option).

## Code availability

The analysis code for this study is available on Open Science Framework (https://osf.io/gd93n).
