# Supplementary material for: Lateralized costs of divided attention to faces
Source: Atten Percept Psychophys. 2025 Dec 28;88(1):38. doi: 10.3758/s13414-025-03189-1 (PMC12745327; doi:10.3758/s13414-025-03189-1)
Supplement: Supplementary file 1 — Supplementary file1 (DOCX 3557 KB) [file 13414_2025_3189_MOESM1_ESM.docx]

**SUPPLEMENTAL MATERIALS**

For *Lateralized costs of divided attention to faces*

**Contents**

[**S.1. Face sex category validation** 2](#_Toc206612089)

[**S.2. Face stimuli** 3](#_Toc206612090)

[**S.3. Judgment-specific face pairs** 3](#_Toc206612091)

[**S.4. Staircase and timing** 4](#_Toc206612092)

[**S.5. Eye-tracking** 5](#_Toc206612093)

[**S.6. A_g_ calculation** 5](#_Toc206612094)

[**S.7. Preliminary analyses on unsplit data** 7](#_Toc206612095)

[**S.7.1. Effect of response order for dual-task trials** 7](#_Toc206612096)

[**S.7.2. ANOVA: *cue* × *location* at each level of *judgment*** 7](#_Toc206612097)

[**S.7.3. Response time analysis** 8](#_Toc206612098)

[**S.8 Congruency analyses** 9](#_Toc206612099)

[**S.8.1 ANOVA: *congruency* × *cue* × *location* at each level of *judgment*** 9](#_Toc206612100)

[**S.8.2 Response time analysis** 10](#_Toc206612101)

[**S.8.3 Congruency indices** 11](#_Toc206612102)

[**S.9. Additional analyses to control for inflated sex judgment accuracy** 12](#_Toc206612103)

**S.1. Face sex category validation**

Before conducting the experimental procedures detailed in the Method section, a group of six independent observers (who did not then participate in the main experimental procedure) performed a face sex category validation task to select the least ambiguous face stimuli in terms of face sex. We first selected 64 of 100 faces from the Psychological Image Collection at Sterling (PICS; pics.stir.ac.uk) that had been used in preliminary studies in the lab. As described in the Method section, we used greyscale images of faces that were forward-facing with neutral expressions, cropped to remove external facial features (e.g., hair and face shape), and equated for mean luminance using the SHINE toolbox in MATLAB (The MathWorks Inc., 2022; Willenbockel et al., 2010). During the face sex validation task, each face appeared at the center of the screen until the observer pressed “f” to indicate that the face appeared female or “m” to indicate that the face appeared male. After each response, the face image was removed from the screen and replaced with a brief (100 ms) checkerboard-scrambled face mask in the same location. Then, the next face was presented in the same location. Each of the 64 faces was presented twice in a random order.

We first assessed agreement between presentations of the same face for each observer. If an observer entered two different responses for one face, that face was flagged as a candidate for removal. The flagged faces were then aggregated across the six observers. Any face for which none of the observers entered incorrect or conflicting responses (35 of 64, 18 female) was chosen for the final stimulus set. Any face for which two or more observers entered conflicting responses (10 of 64) was removed. The final five faces (2 female, 3 male) were randomly selected from the remaining faces for which only one observer entered conflicting responses. The final stimulus set comprised 40 faces (20 female, 20 male) based on inter-observer agreement (Fig. S1A).

**S.2. Face stimuli**


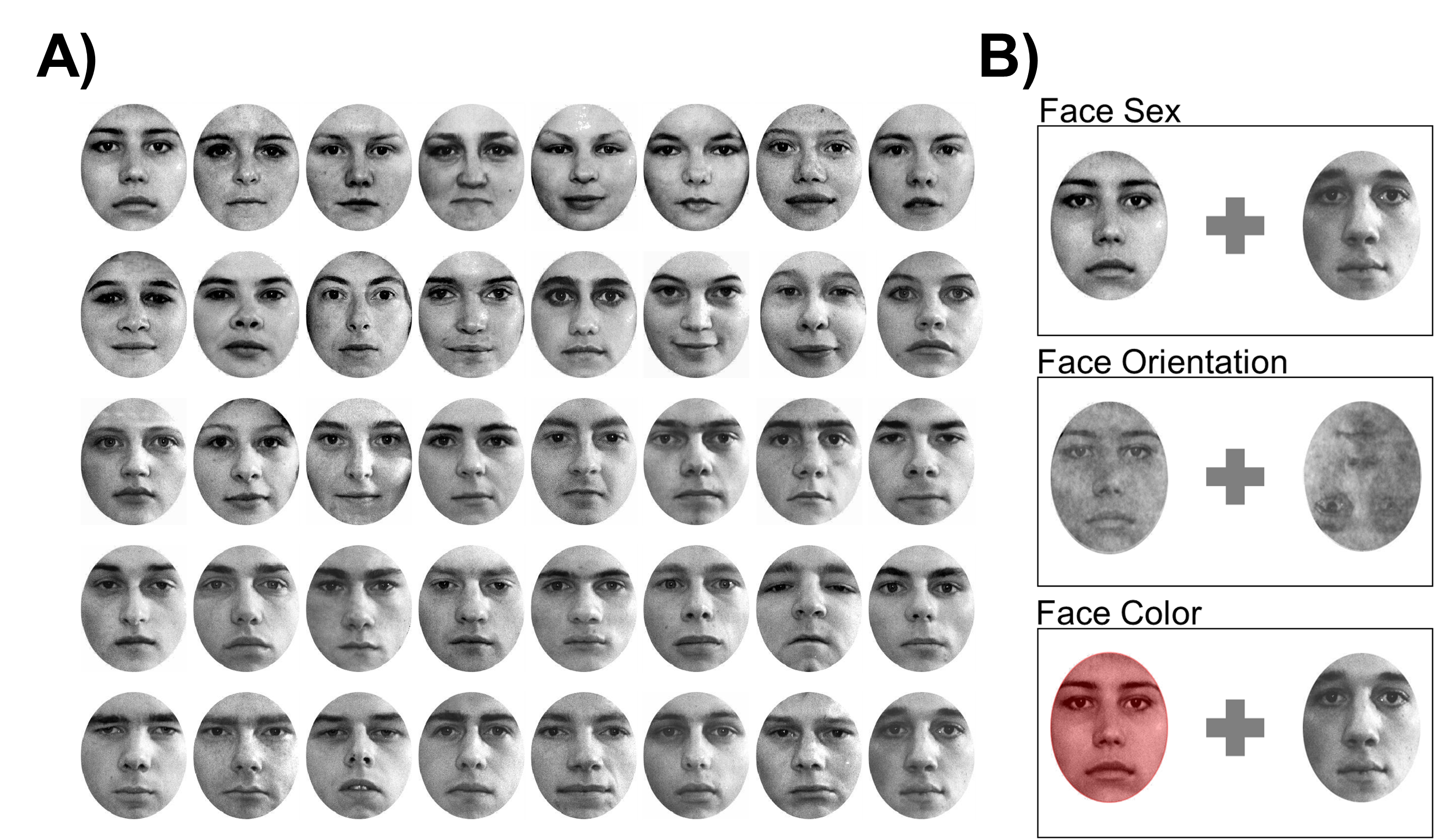


**Figure S1**. Experiment stimuli. (A) Forty face stimuli selected from the Psychological Image Collection at Stirling database (PICS; pics.stir.ac.uk) based on inter-observer agreement in an independent face sex rating task (see S.1. Face sex category validation). The first 20 faces are female and the last 20 faces are male. (B) The same judgment-specific faces pairs as shown in Fig. 1 of the main text, showing red-tint superimposed for the Face Color judgment.

**S.3. Judgment-specific face pairs**

After selection into the stimulus set by an independent group of observers via the face sex category validation task, face stimuli were randomly paired for each *judgment* and for each observer to generate experimental trials. Each face pair comprised two different identities. A key difference between each of the three *judgment* types used here was that each *judgment* was performed on slightly different manipulations of the face pairs (see Fig. 1), though the same face images were used in each (Fig. S1).

As mentioned in the Method, a noteworthy methodological difference between the study reported here and those of previously published work (Campbell et al., 2024; Popovkina et al., 2021, 2023; White et al., 2018, 2020) was the larger number of participants (N = 47, as compared to N ranging from 10 – 12) that were each run through fewer trials. While this approach might have contributed to some of the variability observed between individual participants, a potential strength of this approach is that it might have reduced practice effects related to extensive exposure to each of the 40 faces. Each of the 40 faces appeared 24 times, eight times per *judgment* type: twice when cued to the LVF (once in the cued location and once in the uncued location), twice when cued to the RVF (once in the cued location and once in the uncued location), and four times in the dual-task condition (once in each hemifield for each *cue* order). To test for effects of face repetition across *cue* and *judgment* types, we split experimental trials in half for each participant and measured the accuracy (mean A_g_ ± SEM) in each half. For the sex judgment trials, no difference was observed between mean A_g_ in the first half and mean A_g_ in the second half of the experiment (M_difference_ = -0.002 ± 0.01; *t*(46) = -0.02, *p* = .870, *d* = -0.03). The same was true for the orientation judgment (M_difference_ = -0.02 ± 0.03; *t*(46) = -0.53, *p* = .690, *d* = -0.08) and the color judgment (M_difference_ = -0.02 ± 0.01; *t*(46) = -1.75, *p* = .197, *d* = -0.26).

**S.4. Staircase and timing**

Before starting the main experimental trials, participants completed a separate adaptive staircase procedure for each of the three *judgment* types (i.e., sex, orientation, and color) on a set of single-task trials (Campbell et al., 2024; White et al., 2018, 2020) using the Palamedes Toolbox for MATLAB (Prins & Kingdom, 2018). The staircases used for the sex and color judgments were descending and used a one-up, one-down procedure in which the step size up was three times larger than the step size down. The staircase for the orientation judgment was ascending and used a one-up, one-down procedure in which the step size down was three times larger than the step size up. Each staircase was run in blocks of 20 trials each, and the blocks alternated between each *judgment*-specific category (female or male, upright or inverted, and red-tinted or greyscale).

For the sex judgment, the staircase was used to manipulate the inter-stimulus interval (ISI), or the blank interval between the pre- and post-masks and the face stimuli (see Fig. 2). Participants first completed the staircase for the face sex judgment condition to get the ISI for the remaining two staircase procedures and the main experiment, similar to the procedure used by White et al. (2018). Correct sex judgments decreased the ISI by one step, whereas incorrect responses increased the ISI by one step; the step size up was three times larger than the step size down. The ISI was independently manipulated for each visual field, and the fastest of the two ISIs was then used in subsequent procedures. For the orientation and color judgments, the staircase was used to manipulate the alpha value of the superimposed Gaussian noise mask or color mask, respectively. Alpha ranged from 0 to 1, with 0 being fully transparent and 1 being fully opaque. Correct color judgments decreased the alpha value (thereby increasing transparency) by one step, whereas incorrect color responses increased the alpha value (and thus increased opacity) by one step; again, the step size up was three times as large as the step size down. The lowest alpha value between the two hemifields was then implemented in the main experiment. Correct orientation judgments increased the alpha value by one step and incorrect orientation judgments decreased the alpha value by one step; in this case, the step size down was three times larger than the step size up. The largest alpha value between the two hemifields was then taken for the main experiment. The order of the orientation and color judgment staircases was counterbalanced between participants.

This procedure was implemented to limit stimulus visibility and obtain a mean percent-correct accuracy of approximately 75% for the single-task trials across the three *judgment* types. Participants completed thirteen reversals, or switches between correct and incorrect responses. The first three reversals were removed from analysis, and the ISI and alpha values were taken as the mean threshold value of the last ten reversals from their respective staircase procedure.

**S.5. Eye-tracking**

Participants were instructed to maintain central fixation during each trial. During the staircases and main experimental procedure, fixation breaks were monitored by recording the binocular gaze position of each participant using a GP3 eye-tracker system with a 60 Hz refresh rate (Gazepoint, Vancouver, BC). If the participant’s gaze fell beyond 2.4 degrees horizontally from the fixation cross, the participant was presented with a warning (“Gaze moved too far”) on the screen, and the trial sequence was restarted from the fixation period. For some participants (N = 32), eye tracking was performed but trials were not restarted and were instead manually removed during analysis. Data from these participants were excluded if ten or more trials were removed from any condition of the main experiment (19 of the 32 participants were excluded based on this criterion). An additional six observers were excluded due to an inability to collect adequate eye-tracking data (e.g., due to calibration errors). For the remaining observers (N = 51), an average of 4.5 (SEM = 1.2) trials were removed from analysis due to fixations breaks.

**S.6. A_g_ calculation**

As described in previously published studies, we quantified performance as Green’s area (A_g_), a bias-free measure of accuracy that is calculated as the area under the receiver operating characteristic (ROC) curve (Campbell et al., 2024; Pollack & Hsieh, 1969; Popovkina et al., 2021, 2023; White et al., 2018, 2020). The ROC curve is created using proportion of false alarms (along the x-axis) and proportion of hits (along the y-axis) (Nahm, 2022). The points defining the curve are generated by grouping hits and false alarms differently depending on observer confidence. The group-average ROC curve for the face sex judgment is shown in Fig. S2. To get the leftmost point along the curve, hits and false alarms are only considered as such if the observer was confident (“sure”) in their response. To get the middle point along the curve, hits and false alarms are considered as such regardless of confidence (“sure” or “guess”). Finally, to get the rightmost point along the curve, hits and false alarms are both considered as such regardless of confidence, and false negatives and correct rejections are considered as hits and false alarms, respectively, if the observer was not confident for those responses (“guess”). A line is drawn connecting the three points with (0, 0) and (1, 1), and A_g_ is measured as the proportion of the area beneath the newly created curve. The dashed line connecting (0, 0) and (1, 1) represents an ROC curve if detection was at chance (i.e., 50% area under the curve).


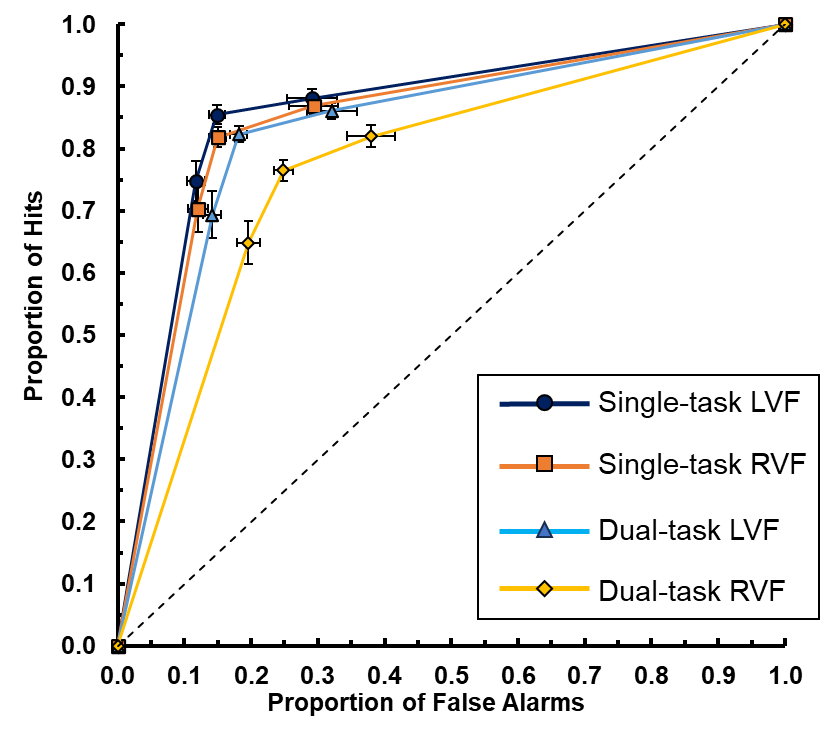


**Figure S2.** The group-average receiver operating characteristic (ROC) curve for judgments of face sex. Each curve represents a cue × location condition. Our reported accuracy measure, A_g_, is calculated for each cue × location condition as the area under each respective curve. Each point along the curve is defined by the proportion of false alarms (x-axis) and the proportion of hits (y-axis), which are both modulated by observer confidence ratings. Error bars represent standard error of the mean.

**S.7. Preliminary analyses on unsplit data**

**S.7.1.** **Effect of response order for dual-task trials**

None of the three *judgment* types showed a significant effect of response order for dual-task trials, in which two responses were entered for each trial. For the sex judgment, the mean difference between response 1 and response 2 A_g_ was -0.001 ± 0.01 (*t*(46) = -0.08, *p* = .932, *d* = -0.01). For the orientation judgment, the mean difference was 0.01 ± 0.01 (*t*(46) = 1.57, *p* = .122, *d* = 0.23). Finally, for the color judgment, the mean difference was -0.004 ± 0.01 (*t*(46) = -0.54, *p* = .592, *d* = -0.08). Because mean A_g_ for response 1 and response 2 did not differ, we collapsed across dual-task response order for each of the three *judgment* types for all analyses.

**S.7.2.** **ANOVA: *cue* × *location* at each level of *judgment***

We conducted three separate 2 (*cue*: single-task, dual-task) × 2 (*location*: LVF, RVF) repeated-measures ANOVAs to assess the potential main effects and interactions of *cue* and *location* at each level of *judgment* (sex, orientation, and color) on single-task and dual-task accuracy (A_g_). Because we used slightly different types of stimulus manipulations in each *judgment* condition, we conducted a separate ANOVA for each level of *judgment* (sex, orientation, and color). Fig. 3A shows mean A_g_ (± standard error of the mean) for all participants (N = 47) across each condition.

**Sex**. For the sex judgment, we observed a significant dual-task cost (i.e., a decrease in mean dual-task A_g_ relative to mean single-task A_g_) and a hemifield asymmetry in the dual-task condition such that mean A_g_ in the LVF was greater than mean A_g_ in the RVF, as evidenced by the following effects: main effects of each of our two conditions (*cue*: *F*(1, 46) = 34.82, *p* < .001, *η^2^_p_* = 0.43; *location*: *F*(1, 46) = 13.51, *p* < .001, *η^2^_p_* = 0.23) and a two-way interaction (*cue* × *location*: *F*(1, 46) = 6.23, *p* = .016, *η^2^_p_* = 0.12). Given the significant two-way interaction, we performed Bonferroni-corrected post-hoc comparisons to further clarify whether statistically significant dual-task costs and hemifield differences occurred. Significant costs were observed in the LVF between single-task and dual-task conditions (M_difference_ = 0.03 ± 0.01; *t*(46) = 3.35, *p* = .009, *d* = 0.49). Larger costs were observed in the RVF between single-task and dual-task conditions (M_difference_ = 0.08 ± 0.02; *t*(46) = 5.21, *p* < .001, *d* = 0.76). While there was no difference between mean A_g_ in the LVF and RVF in the single-task (M_difference_ = 0.02 ± 0.01; *t*(46) = 1.18, *p* = 1.00, *d* = 0.17), an LVF advantage emerged in the dual-task (M_difference_ = 0.06 ± 0.01; *t*(46) = 4.44, *p* < .001, *d* = 0.65).

**Orientation**. As in the sex judgment, we found a modest dual-task cost and evidence of an LVF advantage for the orientation judgment. However, unlike in the sex judgment, the hemifield asymmetry in the orientation judgment did not depend on cue condition: the *cue* × *location* ANOVA yielded main effects of each of our two conditions (*cue*: *F*(1, 46) = 29.19, *p* < .001, *η^2^_p_* = 0.39; *location*: *F*(1, 46) = 10.09, *p* = .003, *η^2^_p_* = 0.18) but no two-way interaction (*F*(1, 46) = 1.42, *p* = .239, *η^2^_p_* = 0.03). There was evidence of a mean dual-task cost, where the mean single-task A_g_ was greater than the mean dual-task A_g_ (M_difference_ = 0.05 ± 0.01). In addition to a dual-task cost, there was also an LVF advantage such that mean LVF A_g_ was greater than mean RVF A­_g_ (M_difference_ = 0.04 ± 0.01).

**Color**. As expected, we saw no evidence of a dual-task cost for the color judgment. Perhaps unexpectedly, however, we found greater A_g_ in the LVF than in the RVF. This was evidenced by a main effect of *location* (*F*(1, 46) = 5.79, *p* = .020, *η^2^_p_* = 0.11) but no effect of *cue* (*F*(1, 46) = 0.67, *p* = .417, *η^2^_p_* = 0.01) and no interaction (*F*(1, 46) = 0.26, *p* = .612, *η^2^_p_* = 0.01). The mean difference between mean A_g_ in the LVF and in the RVF was 0.03 ± 0.01.

**S.7.3. Response time analysis**

Table S1 shows mean response time (RT; ± standard error of the mean) for all participants (N = 47) across each *cue* × *location* condition for each *judgment* type. Median RT for each condition was calculated for each participant, and the medians were then averaged at the group level for analysis. For each of the three *judgment* types, single-task responses were faster than dual-task responses in both the LVF and the RVF.

| **Table S1.** Response time (Mean ± SEM) in each *cue* × *location* condition for each *judgment* type | | | | |
| --- | --- | --- | --- | --- |
| **Judgment** | **Single-task LVF** | **Single-task RVF** | **Dual-task LVF** | **Dual-task RVF** |
| **Sex** | 0.45 ± 0.02 | 0.48 ± 0.03 | 0.66 ± 0.03 | 0.70 ± 0.03 |
| **Orientation** | 0.54 ± 0.03 | 0.56 ± 0.03 | 0.70 ± 0.03 | 0.76 ± 0.04 |
| **Color** | 0.45 ± 0.02 | 0.47 ± 0.03 | 0.62 ± 0.02 | 0.60 ± 0.03 |

**Sex**. RT was faster for the single-task relative to the dual-task condition in the LVF (M_difference_ = -0.21 ± 0.02; *t*(46) = -11.22, *p_Bonferroni_* < .001, *d* = -1.64) and in the RVF (M_difference_ = -0.22 ± 0.02; *t*(46) = -9.96, *p_Bonferroni_* < .001, *d* = -1.45).

**Orientation**. As was observed in the sex judgment, RT in the orientation judgment was faster for the single-task as compared to the dual-task condition in the LVF (M_difference_ = -0.16 ± 0.03; *t*(46) = -6.09, *p_Bonferroni_* < .001, *d* = -0.89) and in the RVF (M_difference_ = -0.20 ± 0.03; *t*(46) = -7.14, *p_Bonferroni_* < .001, *d* = -1.04).

**Color**. Finally, RT for the color judgment was faster for the single-task than for the dual-task condition in the LVF (M_difference_ = -0.17 ± 0.02; *t*(46) = -8.18, *p_Bonferroni_* < .001, *d* = -1.19) and in the RVF (M_difference_ = -0.13 ± 0.02; *t*(46) = -5.26, *p_Bonferroni_* < .001, *d* = -0.77).

**S.8 Congruency analyses**

**S.8.1 ANOVA: *congruency* × *cue* × *location* at each level of *judgment***

We conducted three separate 2 (*congruency*: incongruent, congruent) × 2 (*cue*: single, dual) × 2 (*location*: LVF, RVF) repeated-measures ANOVAs to assess the effects of category-congruency on dual-task cost in each hemifield for each *judgment* type. We again conducted three separate ANOVAs, one for each *judgment* type, due to the slight differences in stimulus manipulations between the *judgment* types. Fig. 5 shows mean A_g_ (± standard error of the mean) for all participants (N = 47) across each condition of interest.

**Sex**. Divided attention and hemifield effects for the sex judgment depended on category-congruency, as evidenced by a three-way interaction between *congruency* × *cue* × *location* (*F*(1, 46) = 12.04, *p* = .001, *η^2^_p_* = 0.21). As such, we next conducted two 2 (*cue*: single, dual) × 2 (*location*: LVF, RVF) repeated-measures ANOVAs, one at each level of *congruency*. When stimuli were incongruent, we observed no dual-task costs and no hemifield asymmetry. That is, we failed to show a main effect of *cue* (*F*(1, 46) = 0.85, *p* = .362, *η^2^_p_* = 0.02), a main effect of *location* (*F*(1, 46) = 0.10, *p* = .749, *η^2^_p_* = 0.002), or an interaction of *cue* × *location* (*F*(1, 46) = 0.50, *p* = .482, *η^2^_p_* = 0.01). When stimuli were congruent, however, we observed dual-task costs that co-occurred with hemifield asymmetry, as evidenced by an interaction between *cue* × *location* (*F*(1, 46) = 12.40, *p* < .001, *η^2^_p_* = 0.21), as well as main effects of *cue* (*F*(1, 46) = 50.80, *p* < .001, *η^2^_p_* = 0.52) and *location* (*F*(1, 46) = 18.70, *p* < .001, *η^2^_p_* = 0.29). Using Bonferroni-correct post hoc tests to clarify the interaction, we found dual-task costs in both the LVF (M_difference_ = 0.05 ± 0.02; *t*(46) = 3.52, *p* = .006, *d* = 0.51) and the RVF (M_difference_ = 0.15 ± 0.02; *t*(46) = 6.23, *p* < .001, *d* = 0.91). While no difference in mean A_g_ was observed between the LVF and the RVF in the single-task condition (M_difference_ = 0.02 ± 0.02; *t*(46) = 1.00, *p* = 1.00, *d* = 0.15), we found an LVF advantage in the dual-task condition (M_difference_ = 0.12 ± 0.02; *t*(46) = 5.61, *p* < .001, *d* = 0.82).

**Orientation**. Divided attention and hemifield effects for the orientation judgment also depended on category-congruency, supported by a three-way interaction between c*ongruency* × *cue* × *location* (*F*(1, 46) = 5.14, *p* = .028, *η^2^_p_* = 0.10). We next conducted two 2 (*cue*: single, dual) × 2 (*location*: LVF, RVF) repeated-measures ANOVAs, one at each level of *congruency*. For incongruent trials, we observed no dual-task costs and no hemifield asymmetries; we failed to show a main effect of *cue* (*F*(1, 46) = 3.52, *p* = .067, *η^2^_p_* = 0.07), a main effect of *location* (*F*(1, 46) = 2.36, *p* = .131, *η^2^_p_* = 0.05), or an interaction between *cue* × *location* (*F*(1, 46) = 0.42, *p* = .518, *η^2^_p_* = 0.01). In contrast, category-congruent stimuli showed evidence of dual-task costs that depended on hemifield *location*, as evidenced by an interaction between *cue* × *location* (*F*(1, 46) = 4.50, *p* = .039, *η^2^_p_* = 0.09), as well as main effects of *cue* (*F*(1, 46) = 34.20, *p* < .001, *η^2^_p_* = 0.43) and *location* (*F*(1, 46) = 9.40, *p* = .004, *η^2^_p_* = 0.17). Using Bonferroni-correct post hoc tests to clarify the interaction, we found dual-task costs in both the LVF (M_difference_ = 0.05 ± 0.02; *t*(46) = 3.02, *p* = .025, *d* = 0.44) and the RVF (M_difference_ = 0.11 ± 0.02; *t*(46) = 5.00, *p* < .001, *d* = 0.73). Again, we found no difference in mean A_g_ between the LVF and RVF in the single-task condition (M_difference_ = 0.02 ± 0.02; *t*(46) = 0.88, *p* = 1.00, *d* = 0.13), but found an LVF advantage in the dual-task condition (M_difference_ = 0.08 ± 0.02; *t*(46) = 3.25, *p* = .013, *d* = 0.47).

**Color**. Finally, the results of the color judgment showed an overall effect of congruency (incongruent mean A_g_ > congruent mean A_g_) and hemifield (mean LVF A_g_ > mean RVF A_g_), but no evidence of a dual-task cost. The ANOVA revealed a main effect of *congruency* (*F*(1, 46) = 86.62, *p* < .001, *η^2^_p_* = 0.66), such that mean A_g_ for category-congruent trials was less than mean A­_g_ for category-incongruent trials (M_difference_ = -0.11 ± 0.01). The ANOVA also revealed a main effect of *location* (*F*(1, 46) = 6.05, *p* = .018, *η^2^_p_* = 0.12), such that mean A_g_ for LVF judgments was greater than mean A_g_ for RVF judgments (M_difference_ = 0.03 ± 0.01). Importantly, however, the ANOVA did not reveal a main effect of *cue* (*F*(1, 46) = 0.61, *p* = .440, *η^2^_p_* = 0.01), nor did it reveal any significant interactions (*p* > 0.50 for all two-way interactions; p = 0.701 for the three-way interaction).

**S.8.2 Response time analysis**

Table S2 shows mean RT (± standard error of the mean) for all participants (N = 47) across each *cue* × *location* condition for each *judgment* type, split by category-congruency (congruent vs. incongruent trials). Median RT for each condition was calculated for each participant, and the medians were then averaged at the group level for analysis.

| **Table S2.** Response time (Mean ± SEM) for each *congruency* × *cue* × *location* condition for each *judgment* | | | | |
| --- | --- | --- | --- | --- |
| **Judgment** | **Single-task LVF** | **Single-task RVF** | **Dual-task LVF** | **Dual-task RVF** |
| **Sex** |  |  |  |  |
| Congruent | 0.47 ± 0.03 | 0.49 ± 0.03 | 0.66 ± 0.03 | 0.72 ± 0.04 |
| Incongruent | 0.47 ± 0.03 | 0.49 ± 0.03 | 0.66 ± 0.03 | 0.69 ± 0.03 |
| **Orientation** |  |  |  |  |
| Congruent | 0.53 ± 0.02 | 0.56 ± 0.03 | 0.68 ± 0.03 | 0.74 ± 0.04 |
| Incongruent | 0.55 ± 0.03 | 0.56 ± 0.03 | 0.73 ± 0.03 | 0.79 ± 0.04 |
| **Color** |  |  |  |  |
| Congruent | 0.46 ± 0.03 | 0.50 ± 0.03 | 0.60 ± 0.02 | 0.58 ± 0.03 |
| Incongruent | 0.44 ± 0.02 | 0.45 ± 0.03 | 0.64 ± 0.03 | 0.63 ± 0.03 |

**Sex**. For the sex judgment, our results showed no difference between congruent and incongruent trials in either the LVF (M_difference_ = 0.004 ± 0.01; *t*(46) = 0.32, *p_Bonferroni_* = 1.00, *d* = 0.05) or in the RVF (M_difference_ = 0.02 ± 0.01; *t*(46) = 1.09, *p_Bonferroni_* = .560, *d* = 0.16).

**Orientation**. For the orientation judgment, congruent responses were faster than incongruent responses in the LVF (M_difference_ = -0.03 ± 0.01; *t*(46) = -2.42, *p_Bonferroni_* = .039, *d* = -0.35), but no difference was apparent in the RVF (M_difference_ = -0.03 ± 0.02; *t*(46) = -1.73, *p_Bonferroni_* = .180, *d* = -0.25).

**Color**. Finally, for the color judgment, no difference was observed between congruent and incongruent responses in either the LVF (M_difference_ = -0.01 ± 0.01; *t*(46) = -0.46, *p_Bonferroni_* = 1.00, *d* = -0.07) or the RVF (M_difference_ = -0.004 ± 0.02; *t*(46) = -0.27, *p_Bonferroni_* = 1.00, *d* = -0.04).

**S.8.3 Congruency indices**

We calculated a *Δ*A_g cong_ index for the congruency cost as the difference between mean congruent and incongruent A_g_ divided by the sum of these values for both the single-task and the dual-task conditions, to allow direct comparison of normalized congruency costs between the different *cue* conditions within each *judgment* type. Fig. S3 shows mean *Δ*A_g cong_ for each *cue* condition across the three *judgment* types. A negative index value indicates greater mean A_g_ for incongruent trials relative to congruent trials. We computed three Bonferroni-correct paired-samples *t*-tests to determine whether the effects of congruency differ between *cue* conditions within each *judgment*. For the sex judgment, mean *Δ*A_g cong_ for the dual-task (M = -0.12 ± 0.01) was less than that of the single-task condition (M = -0.05 ± 0.01), *t*(46) = 5.68, *p* < .001, *d* = 0.82. The same was true for the orientation judgment, in which mean *Δ*A_g cong_ for the dual-task (M = -0.02 ± 0.01) was less than that of the single-task condition (M = 0.01 ± 0.01), *t*(46) = 3.14, *p* = .009, *d* = 0.46. Lastly, for the color judgment, no difference in congruency cost was observed between the single-task (M = -0.08 ± 0.01) and dual-task conditions (M = -0.07 ± 0.01), *t*(46) = -0.33, *p* = 1.00, *d* = -0.05).

We observed congruency costs for judgments of face sex and color in both the single- and the dual-task conditions, and only in the dual-task condition for judgments of face orientation. We implemented three Bonferroni-corrected one-sample *t*-tests to compare congruency cost magnitude relative to 0 for the single-task condition. For both the sex (*t*(46) = -6.29, *p* < .001, *d* = -0.92) and color judgments (*t*(46) = -7.05, *p* < .001, *d* = -1.03), the congruency cost was less than 0 such that there was a significant incongruency advantage. However, the single-task congruency cost for the orientation judgment (*t*(46) = 1.42, *p* = .489, *d* = 0.21) did not differ from 0. Finally, using three Bonferroni-correct one-sample *t*-tests, we compared *Δ*A_g cong_ in the dual-task condition for each *judgment* type relative to 0. Now, judgments of face sex (*t*(46) = -13.20, *p* < .001, *d* = -1.93), color (*t*(46) = -7.84, *p* < .001, *d* = -1.14), and orientation (*t*(46) = -2.57, *p* = .040, *d* = -0.38) each showed congruency costs such that *Δ*A_g cong_ < 0.


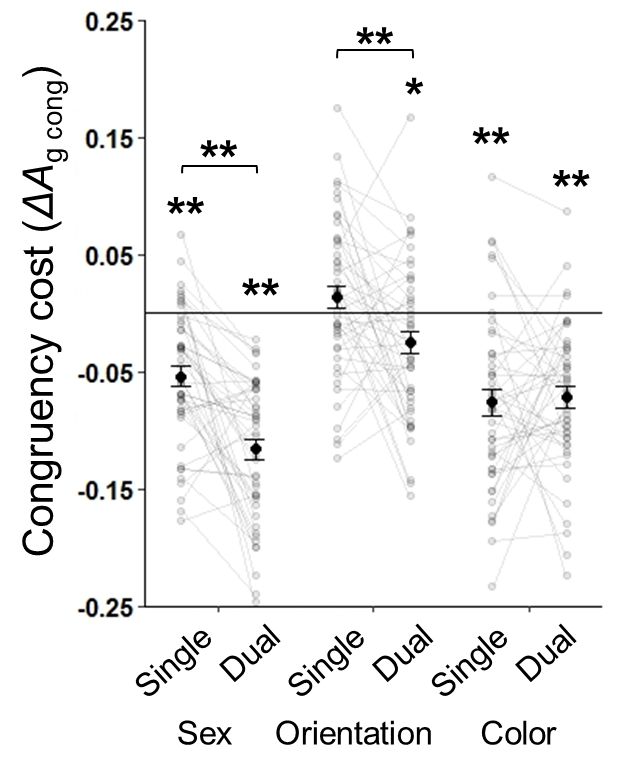


**Figure S3.** Effect of *judgment* type and *cue* on congruency cost (*Δ*A_g cong_). Black points represent the mean *Δ*A_g cong_ (± SEM) at the group level, and grey points (connected by grey lines) represent individual participant results. Negative *Δ*A_g cong_ values indicate greater performance (A_g_) for category-incongruent trials relative to category-congruent trials. Judgments of face sex and color (but not orientation) showed congruency costs for single-task judgments. All three judgment types showed congruency costs for dual-task judgments. Both face-based judgments (sex and orientation) showed greater congruency cost for dual-task judgments as compared to single-task judgments, whereas color judgments showed no difference. Statistically significant differences indicated by asterisks for *p* < 0.05 (*) and for *p* < 0.01 (**) for both one-sample *t*-tests (asterisks directly above individual data points) and for paired-samples *t*-tests (asterisks above horizontal bars).

**S.9. Additional analyses to control for inflated sex judgment accuracy**

As noted in the Results, accuracy (A_g_) was higher for the sex judgment than for the orientation and color judgments. This may be due in part to the elevated A_g_ for incongruent trials relative to congruent trials for judgments of face sex, as shown in Fig. 5. Of primary interest was whether this elevated A_g_ had an effect on the reported lateralized dual-task costs (as shown in Fig. 6). To test the reliability of this effect, we implemented a stricter exclusion criterion on our dataset: we removed any observer for which average single-task and/or dual-task A_g_ was greater than 0.9 for incongruent trials, leaving data from 16 observers for analysis. We collected data from additional participants in the sex categorization task (N = 69) and, using the same exclusion criteria (leaving N = 24), performed the same set of analyses from our main experiment on the combined accuracy-controlled dataset (N = 40).

**ANOVA**. As in S.7.2, we conducted a 2 (*cue*: single-task, dual-task) × 2 (*location*: LVF, RVF) repeated-measures ANOVA to assess the potential main effects and interactions of *cue* and *location* on single-task and dual-task accuracy (A_g_). For the accuracy-controlled sex judgment data, we observed a significant dual-task cost such that single-task A_g_ (M = 0.81 ± 0.01) was greater than dual-task A_g_ (M = 0.73 ± 0.01), as evidenced by a main effect of *cue* (*F*(1, 39) = 61.16, *p* < .001, *η^2^_p_* = 0.61), as shown in Fig. S4A. We also observed a hemifield asymmetry such that mean A_g_ in the LVF (M = 0.79 ± 0.01) was greater than mean A_g_ in the RVF (M = 0.74 ± 0.01), as evidenced by a main effect of *location* (*F*(1, 39) = 14.84, *p* < .001, *η^2^_p_* = 0.28). We did not find a significant interaction between *cue* × *location* (*F*(1, 39) = 1.50, *p* = .228, *η^2^_p_* = 0.04).


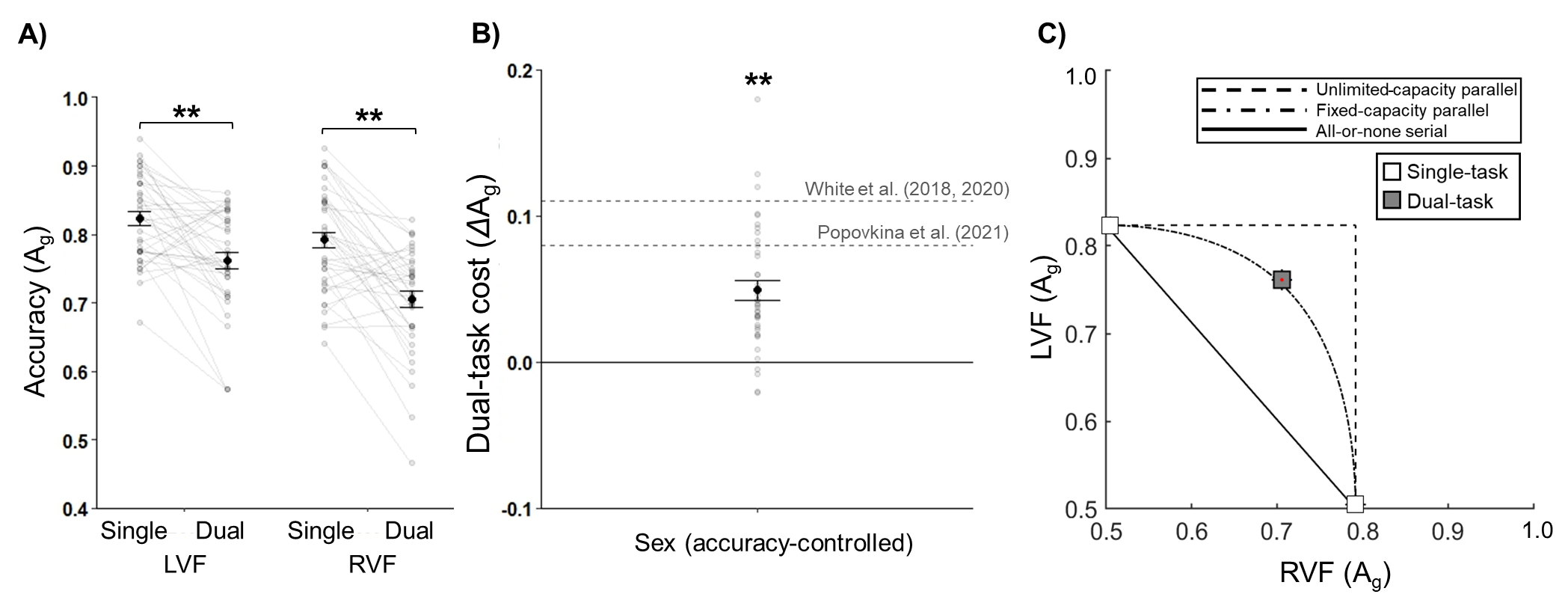


**Figure S4**. Results for accuracy-controlled sex judgment data. (A) Accuracy (Mean A_g_ ± SEM) for each cue × location condition. Mean A_g_ was higher in the single-task condition than in the dual-task condition (** indicates p < 0.01 for the F-test). (B) Effect of judgment type on mean dual-task cost (ΔA_g_). Larger ΔA_g_ values indicate decreased accuracy (A_g_) for dual-task trials relative to single-task trials. Dashed light grey lines represent estimated cost index values derived from A_g_ values reported in Popovkina et al. (2021) and White et al. (2018, 2020) (** indicates p < 0.01 for a one-sample t-test). Black points represent the mean A_g_ (± SEM) in (A) or mean ΔA_g_ (± SEM) in (B) at the group level, and grey points (connected by grey lines) represent individual participant results. (C) Attention Operating Characteristic (AOC). The AOC shows the dual-task cost as dual-task performance (grey squares) relative to the single-task performance (unfilled squares pinned to axes). The results showed a larger cost than would be expected in the unlimited-capacity parallel model and instead showed a modest cost best fit by the fixed-capacity parallel model (dot-dashed curve). There was no evidence of all-or-none serial processing (solid diagonal line). Error bars represent standard error of the mean.

**Dual-task cost index**. As was done for the main experiment, we calculated a *Δ*A_g_ index for the dual-task cost as the difference between mean single- and dual-task A_g_ divided by the sum of these values. Fig. S4B shows mean *Δ*A_g_ for the accuracy-controlled sex judgment data. A one-sample *t*-test showed that *Δ*A_g_ > 0 (M = 0.05 ± 0.01, *t*(39) = 7.37, *p* < .001, *d* = 1.16). Approximate cost indices (represented by dashed light grey lines in Fig. S4B) were estimated from single- and dual-task A_g_ values reported by White et al. (2018, 2020; *Δ*A_g_ = 0.11) and Popovkina et al. (2021; *Δ*A_g_ = 0.08). The costs reported here were about 63% of those reported by Popovkina et al. and 45% of those reported by White et al.

**Attention Operating Characteristic (AOC)**. As shown in Fig. S4C, the results of the accuracy-controlled sex judgment data fall on the fixed-capacity parallel model curve. The mean distance from the all-or-none serial line was 0.11 ± 0.01, which was significantly greater than 0 (one-sample *t*(39) = 9.05, *p_Bonferroni_* < .001, d = 1.43). The distance from the fixed-capacity curve was 0.02 ± 0.01, which did not differ from 0 (one-sample *t*(39) = 1.40, *p_Bonferroni_* = .337, d = 0.22). Additionally, the magnitude of the cost (M = 0.07 ± 0.01) was larger than that predicted by the unlimited capacity parallel model (one-sample *t*(39) = 7.82, *p* < .001, d = 1.24). We take this as evidence that the result of the accuracy-controlled sex judgment data was best predicted by the fixed-capacity parallel model.

**Congruency effects**. We conducted a 2 (*congruency*: incongruent, congruent) × 2 (*cue*: single, dual) × 2 (*location*: LVF, RVF) repeated-measures ANOVA to assess the effects of category-congruency on single- and dual-task A_g_ in each hemifield. Divided attention and hemifield effects for the accuracy-controlled sex judgment data depended on category-congruency, as evidenced by a three-way interaction between *congruency* × *cue* × *location* (*F*(1, 39) = 11.70, *p* = .001, *η^2^_p_* = 0.23). As such, we next conducted two 2 (*cue*: single, dual) × 2 (*location*: LVF, RVF) repeated-measures ANOVAs, one at each level of *congruency*.

As shown in Fig. S5A (left panel), when stimuli were incongruent, we observed no dual-task costs and no hemifield asymmetry. We did not observe a main effect of *cue* (*F*(1, 39) = 0.05, *p* = .827, *η^2^_p_* = 0.001) or of *location* (*F*(1, 39) = 2.57, *p* = .117, *η^2^_p_* = 0.06). While the interaction of *cue* × *location* was significant (*F*(1, 39) = 4.20, *p* = .047, *η^2^_p_* = 0.10), Bonferroni-correct post-hoc tests did not reveal costs in the LVF (M_difference_ = 0.02 ± 0.02; *t*(39) = 1.13, *p* = 1.00, *d* = 0.18) or the RVF (M_difference_ = -0.03 ± 0.01; *t*(39) = -1.87, *p* = .415, *d* = -0.23). Mean A_g_ did not differ between the LVF and RVF in either the single-task condition (M_difference_ = 0.04 ± 0.02; *t*(39) = 2.02, *p* = .303, *d* = 0.32) or in the dual-task condition (M_difference_ = -0.01 ± 0.01; *t*(39) = -0.55, *p* = 1.00, *d* = -0.09).

As shown in Fig. S5A (right panel), when stimuli were congruent, we observed dual-task costs that co-occurred with hemifield asymmetry, as evidenced by an interaction between *cue* × *location* (*F*(1, 39) = 8.00, *p* = .007, *η^2^_p_* = 0.17), as well as main effects of *cue* (*F*(1, 39) = 131.75, *p* < .001, *η^2^_p_* = 0.77) and *location* (*F*(1, 39) = 14.10, *p* < .001, *η^2^_p_* = 0.26). Using Bonferroni-correct post hoc tests to clarify the interaction, we found dual-task costs in both the LVF (M_difference_ = 0.10 ± 0.02; *t*(39) = 5.06, *p* < .001, *d* = 0.80) and in the RVF (M_difference_ = 0.20 ± 0.02; *t*(39) = 9.12, *p* < .001, *d* = 1.44). While there was no difference in mean A_g_ between the LVF and the RVF in the single-task condition (M = 0.02 ± 0.02; *t*(39) = 0.84, *p* = 1.00, *d* = 0.13), we found an LVF advantage in the dual-task condition (M = 0.11 ± 0.02; *t*(39) = 4.73, *p* < .001, *d* = 0.75).

**
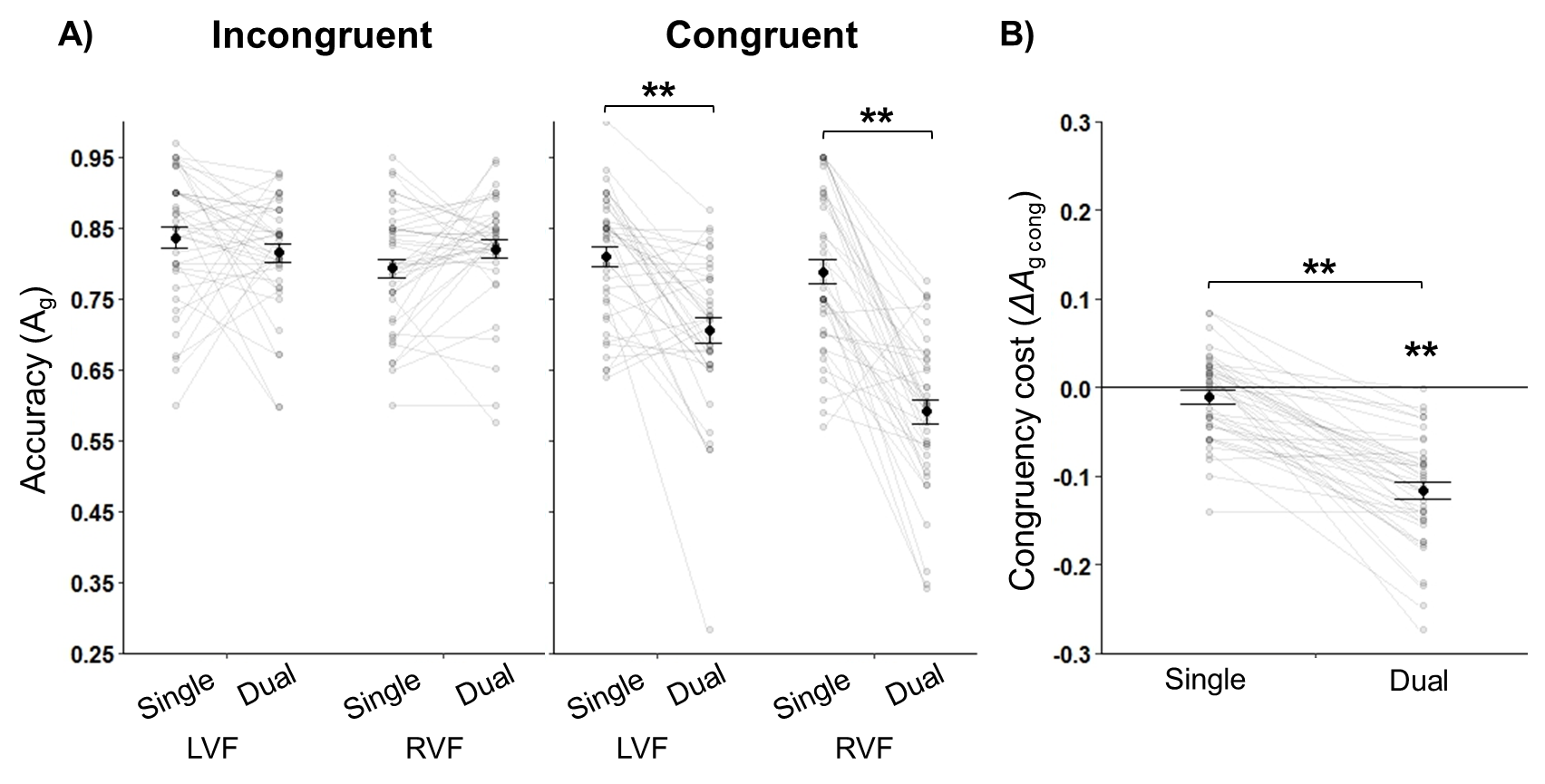
**

**Figure S5**. Results of the congruency analyses for the accuracy-controlled sex judgment data. Black points represent the mean A_g_ (± SEM) in (A) or mean ΔA_g cong_ (± SEM) in (B) at the group level, and grey points (connected by grey lines) represent individual participant results. (A) Accuracy (A_g_) results split by congruency (incongruent, left; congruent, right). Single-task A_g_ did not differ from dual-task A_g_ when stimuli were category-incongruent. In contrast, mean A_g_ was higher in the single-task condition than in the dual-task condition when face stimuli were category-congruent (** indicates p < 0.01 for paired-samples t-tests). (B) Effect of cue on congruency cost (ΔA_g cong_). Larger ΔA_g cong_ magnitudes indicate greater performance (A_g_) for category-incongruent trials relative to category-congruent trials. Accuracy-controlled judgments of face sex showed congruency costs for dual-task judgments but not for single-task judgments. Statistically significant differences indicated by asterisks for p < 0.01 (**) for both one-sample t-tests (asterisks directly above individual data points) and paired-samples t-tests (asterisks above horizontal bars).

**Congruency indices**. We next calculated a *Δ*A_g cong_ index for congruency cost as the difference between mean congruent and incongruent A_g_ divided by the sum of these values for both the single-task and the dual-task conditions. Fig. S5B shows mean *Δ*A_g cong_ for each *cue* condition. A larger index magnitude indicates greater mean A_g_ for incongruent trials relative to congruent trials. We computed a paired-samples *t*-test to determine whether the effects of congruency differ between *cue* conditions. Mean *Δ*A_g cong_ for the dual-task (M = -0.12 ± 0.01) was of greater magnitude than that of the single-task condition (M = -0.01 ± 0.01), *t*(39) = 9.23, *p* < .001, *d* = 1.46. Unlike the results reported in the main experiment, the accuracy-controlled sex judgment data showed an effect of congruency in the dual-task condition (*t*(39) = -11.91, *p* < .001, *d* = -1.88) but not in the single-task condition (*t*(39) = -1.28, *p* = .416, *d* = -0.20) using Bonferroni-correct one-sample *t*-tests relative to a congruency cost of 0.

**LVF advantage**. Finally, we assessed the effect of congruency and *cue* on hemifield asymmetry by examining the difference between mean A_g_ for LVF and RVF judgments in incongruent and congruent trials. We calculated another *Δ*A_g_ index for dual-task cost as the difference between mean single- and dual-task A_g_ divided by the sum of these values for each *location* (LVF/RVF) for each type of category-congruency (incongruent/congruent). Fig. S6 shows mean *Δ*A_g_ for each type of category-congruency at each *location*. We then conducted a 2 (*congruency*: incongruent, congruent) × 2 (*location*: LVF, RVF) repeated-measures ANOVA comparing the normalized *Δ*A_g_ index values.


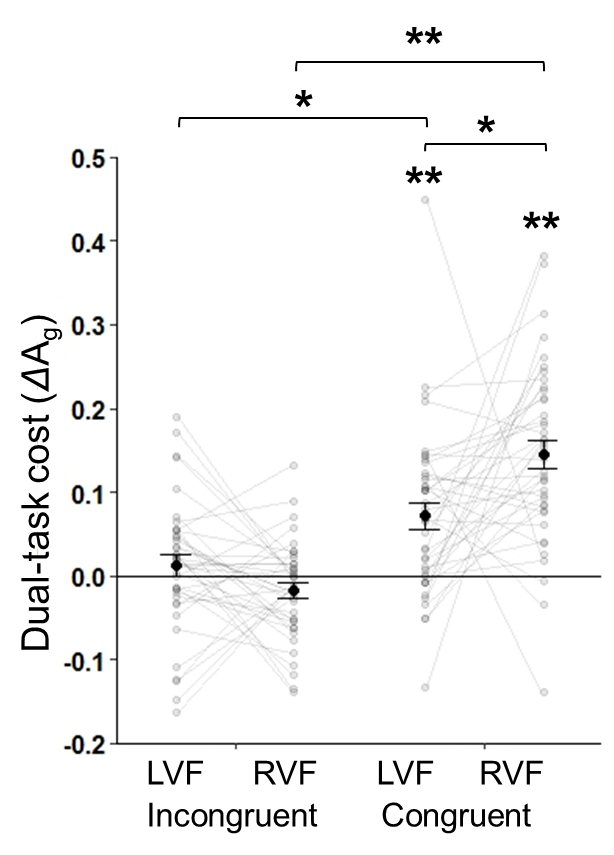


**Figure S6**. Effect of stimulus congruency and cue on dual-task cost and hemifield cost asymmetries (as in Fig. S4, larger ΔA_g_ values indicate decreased dual-task A_g_ relative to single-task A_g_). Black points represent the mean ΔA_g_ (± SEM) at the group level, and grey points (connected by grey lines) represent individual participant results. No dual-task costs or hemifield cost asymmetries were observed for incongruent trials. For congruent trials, dual-task costs were greater than 0 for both LVF and RVF judgments. An LVF cost advantage was apparent for congruent trials, such that the cost in the RVF was greater than the cost in the LVF. Statistically significant differences indicated by asterisks for p < 0.05 (*) and for p < 0.01 (**) for both one-sample t-tests (asterisks directly above individual data points) and for one-tailed paired-samples t-tests (asterisks above horizontal bars).

Like in the results of the main experiment, dual-task costs for the accuracy-controlled sex judgment data were largely driven by a cost in the RVF when stimuli were congruent. The effect of *location* depended on stimulus *congruency*, as evidenced by a two-way interaction (*F*(1, 39) = 11.37, *p* = .002, *η^2^_p_* = 0.23). While the main effect of *congruency* was significant (*F*(1, 39) = 80.46, *p* < .001, *η^2^_p_* = 0.67), the main effect of *location* was not (*F*(1, 39) = 2.39, *p* = .130, *η^2^_p_* = 0.06). As in the primary analysis, we next conducted four planned one-tailed paired-samples *t*-tests with Bonferroni correction. The first pair of tests tested for LVF-RVF differences within the incongruent and the congruent trials. No LVF-RVF difference was found for incongruent trials (M_difference_ = -0.03 ± 0.02; *t*(39) = -1.96, *p* = .113, *d* = -0.31), but a difference emerged for congruent trials such that dual-task cost in the RVF was greater than cost in the LVF (M_difference_ = 0.07 ± 0.02; *t*(39) = 2.89, *p* = .012, *d* = 0.46). The second pair of *t*-tests determined whether costs differed between types of category-congruency by comparing costs between incongruent and congruent trials at the same *location* (i.e., LVF-LVF and RVF-RVF). Costs were greater for congruent trials as compared to incongruent trials in both the LVF (M_difference_ = 0.06 ± 0.02; *t*(39) = 2.86, *p* = .014, *d* = 0.45) and the RVF (M_difference_ = 0.16 ± 0.02; *t*(39) = 8.92, *p* < .001, *d* = 1.41).

Bonferroni-corrected two-tailed, one-sample *t*-tests used to determine whether *Δ*A_g_ > 0 in each hemifield revealed significant dual-task costs in both the LVF (*t*(39) = 4.56, *p* < .001, *d* = 0.72) and RVF (*t*(39) = 8.59, *p* < .001, *d* = 1.36) for congruent trials, but no such cost for incongruent trials (LVF: *t*(39) = 0.99, *p* = 1.00, *d* = 0.16; RVF: *t*(39) = -1.86, *p* = .282, *d* = -0.29).

**REFERENCES**

Campbell, M., Oppenheimer, N., & White, A. L. (2024). Severe processing capacity limits for sub-lexical features of letter strings. *Attention, Perception, & Psychophysics*, *86*(2), 643–652. https://doi.org/10.3758/s13414-023-02830-1

Nahm, F. S. (2022). Receiver operating characteristic curve: Overview and practical use for clinicians. *Korean Journal of Anesthesiology*, *75*(1), 25–36. https://doi.org/10.4097/kja.21209

Pollack, I., & Hsieh, R. (1969). Sampling variability of the area under the ROC-curve and of d’e. *Psychological Bulletin*, *71*(3), 161–173. https://doi.org/10.1037/h0026862

Popovkina, D. V., Palmer, J., Moore, C. M., & Boynton, G. M. (2021). Is there a serial bottleneck in visual object recognition? *Journal of Vision*, *21*(3), 15. https://doi.org/10.1167/jov.21.3.15

Popovkina, D. V., Palmer, J., Moore, C. M., & Boynton, G. M. (2023). Testing hemifield independence for divided attention in visual object tasks. *Journal of Vision*, *23*(13), 3. https://doi.org/10.1167/jov.23.13.3

Prins, N., & Kingdom, F. A. A. (2018). Applying the Model-Comparison Approach to Test Specific Research Hypotheses in Psychophysical Research Using the Palamedes Toolbox. *Frontiers in Psychology*, *9*, 1250. https://doi.org/10.3389/fpsyg.2018.01250

The MathWorks Inc. (2022). *MATLAB* (Version 9.13.0 (R2022b)) [Computer software]. The MathWorks Inc. https://www.mathworks.com

White, A. L., Palmer, J., & Boynton, G. M. (2018). Evidence of Serial Processing in Visual Word Recognition. *Psychological Science*, *29*(7), 1062–1071. https://doi.org/10.1177/0956797617751898

White, A. L., Palmer, J., & Boynton, G. M. (2020). Visual word recognition: Evidence for a serial bottleneck in lexical access. *Attention, Perception, & Psychophysics*, *82*(4), 2000–2017. https://doi.org/10.3758/s13414-019-01916-z

Willenbockel, V., Sadr, J., Fiset, D., Horne, G. O., Gosselin, F., & Tanaka, J. W. (2010). Controlling low-level image properties: The SHINE toolbox. *Behavior Research Methods*, *42*(3), 671–684. https://doi.org/10.3758/BRM.42.3.671
